# Solid state ionics enabled ultra-sensitive detection of thermal trace with 0.001K resolution in deep sea

**DOI:** 10.1038/s41467-022-35682-8

**Published:** 2023-01-12

**Authors:** Yucheng Zhang, Dekai Ye, Mengxue Li, Xi Zhang, Chong-an Di, Chao Wang

**Affiliations:** 1 Key Lab of Organic Optoelectronics & Molecular Engineering, Department of Chemistry, Tsinghua University, Beijing, 100084 China; 2 Beijing National Laboratory for Molecular Sciences, CAS Key Laboratory of Organic Solids, Institute of Chemistry, Chinese Academy of Sciences, Beijing, 100190 China; 3 Zhangjiang Laboratory, 100 Haike Road, Shanghai, 201210 China

**Keywords:** Gels and hydrogels, Sensors and biosensors, Thermoelectrics

## Abstract

The deep sea remains the largest uncharted territory on Earth because it’s eternally dark under high pressure and the saltwater is corrosive and conductive. The harsh environment poses great difficulties for the durability of the sensing method and the device. Sea creatures like sharks adopt an elegant way to detect objects by the tiny temperature differences in the seawater medium using their extremely thermo-sensitive thermoelectric sensory organ on the nose. Inspired by shark noses, we designed and developed an elastic, self-healable and extremely sensitive thermal sensor which can identify a temperature difference as low as 0.01 K with a resolution of 0.001 K. The sensor can work reliably in seawater or under a pressure of 110 MPa without any encapsulation. Using the integrated temperature sensor arrays, we have constructed a model of an effective deep water mapping and detection device.

## Abstract (web summary)

Exploration of deep sea areas relies on sonar and other visual and auditory sensors, but gaining information via temperature sensing in deep sea areas is difficult to realize. Here, the authors design an elastic, self-healable and sensitive thermal sensor which can identify a temperature difference as low as 0.01 K with a resolution of 0.001 K

## Introduction

More than 80% of the ocean remains unexplored due to darkness, high pressure, and corrosive and conductive saltwater. Active sonar technology is the most effective technology in sea exploration^1^. However, active sonar equipment will significantly affect and even kill marine lives, and there are limited power supplies available for high-energy sound waves in the deep sea. Hence, active sonar cannot be used for long periods of time. And the information from sound waves does not reflect the difference between solids, such as a submarine sinking in a pile of rocks^2^. In terms of materials, traditional electronic materials (mostly corrodible and brittle) need bulky, heavy encapsulation to tolerate the high pressure in deep sea as well as the salty nature of sea water. It is therefore important to develop technologies for deep sea exploration.

**Fig. 1:**
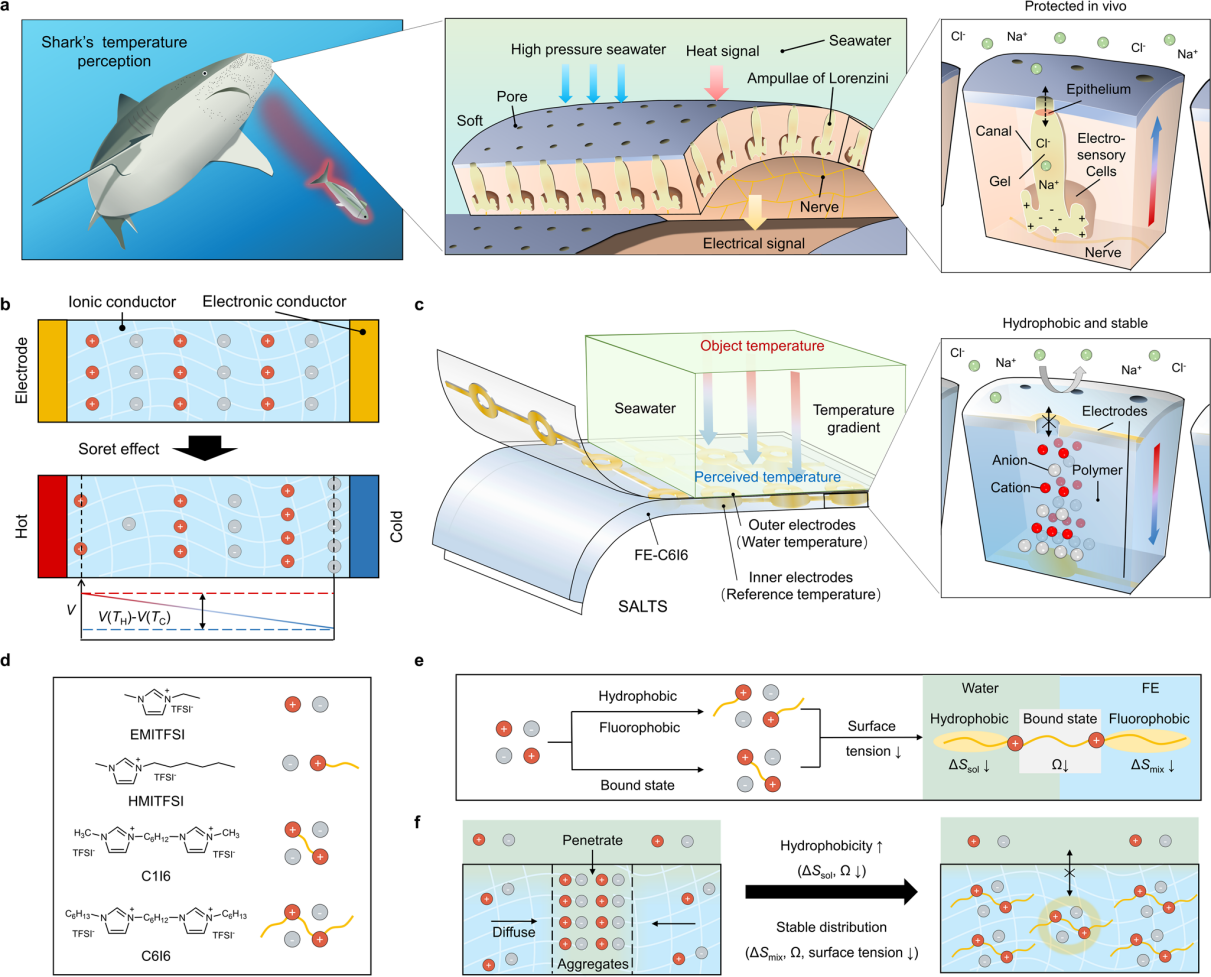
Mechanism of Shark’s temperature perception and design of the artificial sensor consisting of an underwater stable ionic conductor. **a** Sharks tracking prey. The middle inset shows the arrangement of AoL. The bottom inset shows the morphology and composition of AoL. The salt concentration of the gel is the same as that of seawater reducing the osmotic pressure between the inside and outside. The epithelium further isolates the gel from seawater, allowing little interference with ion movement in the gel. **b** Mechanism of voltage generation based on Soret effect. **c** The artificial sensor, SALTS. The inset shows the structure of a single site. Because of the hydrophobic and stable nature, internal ion movement is completely undisturbed by seawater. **d** Chemical structures of the ionic liquids studied. TFSI^-^, bis(trifluoromethanesulphonyl)imide anion. **e** Strategies for designing a highly hydrophobic ionic liquid with thermodynamic stability in FE. Δ*S*_sol_, dissolution entropy in water. Δ*S*_mix_, entropy of ionic liquids mixed with FE. *Ω*, the number of the microstates. **f** Relationship between the properties of ionic liquids and the underwater stability of materials.

In addition to the visual and auditory functions that sonar brings, temperature is equally important in the deep sea, giving us a lot of information about heat. The Soret effect is a special thermoelectric phenomenon that occurs in certain ionic gels. Under a temperature gradient, the difference in thermo-diffusive abilities between cations and anions will generate a concentration gradient, leading to a voltage difference^3–5^ (Fig. 1b). The Seebeck coefficient is defined as ${S}_{{{{{{\rm{t}}}}}}}=-\frac{V\left({T}_{{{{{{\rm{H}}}}}}}\right)-V({T}_{{{{{{\rm{C}}}}}}})}{{T}_{{{{{{\rm{H}}}}}}}-{T}_{{{{{{\rm{C}}}}}}}}$. $V({T}_{{{{{{\rm{H}}}}}}})$ and $V({T}_{{{{{{\rm{C}}}}}}})$ are the voltages of hot electrode at ${T}_{{{{{{\rm{H}}}}}}}$ temperature and cold electrode at ${T}_{{{{{{\rm{C}}}}}}}$ temperature. Sharks have a gel-rich organ, ampullae of Lorenzini (AoL). The ultra-sensitive (~0.3 mV/K) AoL organ based on Soret effect is predicted to detect a tiny temperature variation as little as 0.001 K^6^. And hundreds or even thousands of AoLs in sharks’ snouts can actively detect spatial distribution of temperature in seawater, i.e. heat traces (Fig. 1a). However, the sensitivity of the best man-made temperature sensors based on resistance temperature detector (RTD) is still one order of magnitude behind shark nose. Moreover, conventional sensing materials are not designed to accommodate high pressure seawater. Hence, learning from nature, we report a self-healing, aquatic-stable and ultrasensitive thermal sensor (SALTS) based on the Soret effect for deep sea exploration.

To enable the detection of underwater heat traces, we are motivated to design a device that can match or outperform AoLs. The central step towards this goal is the design of ionic thermoelectric materials for stable and sensitive signals underwater. Fortunately, most of them have the same or higher Seebeck coefficients as AoLs^7–11^. Besides, they have soft structures similar to the gels in AoLs^12,13^, and biomimetic soft structures are expected to live with deep sea environments^14^. However, ionic thermoelectric materials also have their own problems underwater. Due to hygroscopic nature or water solubility of ions, most of the ionic thermoelectric materials can’t maintain desired mechanical and thermoelectric properties^13,15–18^. The water environment that exists within the material can be linked to the outside and lead to signal interference from external factors (movement of water, Na^+^ and Cl^−^, etc.). Meanwhile, to measure tiny temperature changes in water, the material should be in contact with the water as much as possible. Therefore, even without shark’s intricate physiology^12^, the material exposed to the deep sea should be guaranteed to perform consistently. The ionic conductor used to make an artificial snout should satisfy the following requirements: (i) electrical stability based on internal ion movement isolated from external seawater; (ii) excellent mechanical properties that can resist damage in the deep sea. Such materials can directly sense temperature in deep sea (Fig. 1c).

## Results

For SALTS, we designed and developed a composite consisted of a fluoroelastomer (poly(vinylidene fluoride-co-hexafluoropropylene) (FE) and a specially designed hydrophobic ionic liquid with bis(trifluoromethanesulphonyl)imide anion (TFSI^−^) (Fig. 1d, Supplementary Information). Different from conventional ionic liquids, we designed and synthesized C6I6, a gemini-type ionic liquids that are linked by side chains and the positive charges are also protected by alkyl chains. This structure allows C6I6 in FE to meet the characteristics of stable particles in colloidal systems: low diffusion (Brownian motion), and low surface tension. Therefore, drawing on the strategy of stable colloids, C6I6 have a stable distribution in FE to resists seawater penetration and its own leakage. Meanwhile, the alkyl linker and side chains also make C6I6 much more hydrophobic. In this way, FE-C6I6 composite (the mass ratio of FE to C6I6 is 2:1) has stable mechanical and thermoelectric properties (−0.51 mV/K) in seawater. The sensor possesses a responsiveness as low as 0.01 K and a resolution of 0.001 K. It can stably work in seawater without any encapsulations. Notably, the thermoelectric sensor can sustain an extremely high pressure of 110 MPa without any decay in performances, thanks to its self-healing properties and the excellent stability of the composite. Moreover, the sensor arrays can detect spatial distribution of temperature in seawater. Such characteristics allow the sensor to be exposed to the deep sea for accurate temperature detection.

**Fig. 2:**
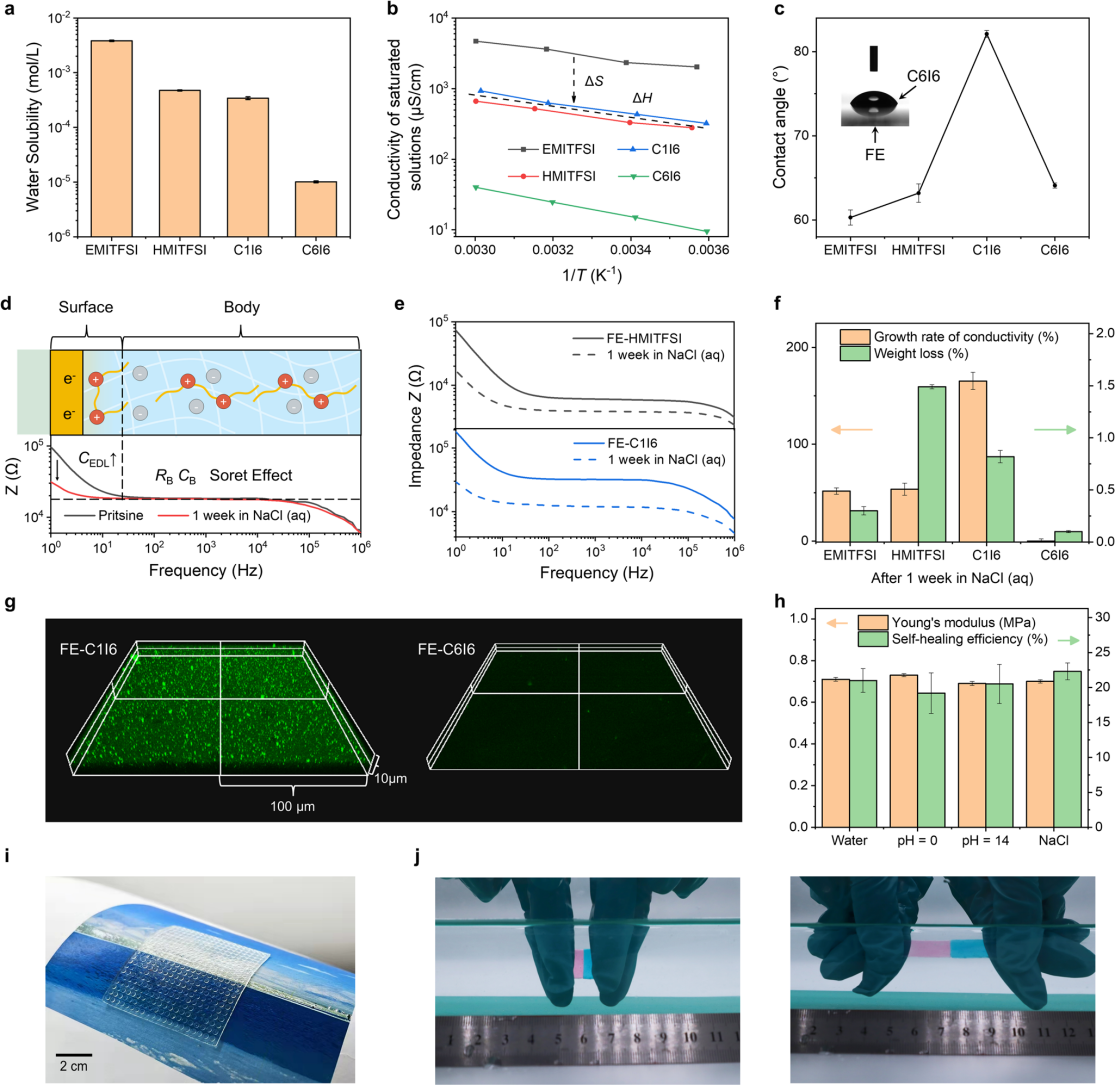
Manifestation and mechanism of the ultra-high underwater stability. **a** Water solubility calculated from the conductivity of a saturated solution containing an ionic liquid. Ambient temperature, 20 °C. Error bars show s.d., *n* = 3. **b** Linear fitting of conductivity of saturated solutions (proportional to the number of charges in solutions after temperature compensation) to temperature (*T*). The intercept becomes proportional to the entropy (Δ*S*) generated by the dissolution-diffusion process (Supplementary Note). **c** Contact angle test with an ionic liquid droplet (2 μL) on bare FE. Error bars show s.d., *n* = 3. **d** Bode plots obtained from the original FE-C6I6 sample and the sample immersed in seawater for a week. The top image shows the main structures corresponding to the different parts of the curve (Supplementary Note). *Z*, impedance. *C*_EDL_, capacitance of electrical double layer. *R*_B_, bulk resistance (ion resistance). *C*_B_, bulk capacitance (geometrical capacitance). **e** Bode plots obtained from the other samples. **f** Growth change of the conductivity and weight loss of the sample immersed in seawater for a week. Error bars show s.d., *n* = 3. **g** LSCM images of the sample immersed in a 10 mmol fluorescein solution for a week. Magnification, 60x. **h** Young’s moduli of the composites immersed in solutions for a week and self-heling efficiencies after 12 h immersed in solutions. Error bars show s.d., *n* = 3. **i** Photograph of a large transparent FE-C6I6 with an array of micropillars on the surface. **j** Photographs of a self-healed sample being stretched to about 3 times. **d**–**j** The mass ratio of FE to ionic liquids is 2:1.

To verify our design, we first tested the water solubility of pure ionic liquids by measuring the conductivity of saturated aqueous solutions. Compared with other hydrophobic ionic liquids which has a hygroscopic nature^19^, C6I6 is highly hydrophobic with a water solubility as low as 10 μmol/L (Fig. 2a). Moreover, the relation between conductivity of saturated ionic liquid solutions and temperature shows that C6I6 has the lowest entropy (intercept in Fig. 2b) owing to its structure (Supplementary Note). The linker contributes to the reduction in the number of microstates (*Ω*) because of conversion from two cations to a single dication^20^. And the hydrophobic side chain contributes to the reduction in the entropy of dissolution (Δ*S*_sol_)^21^. Entropy effects can also be observed in C6I6 mixed with FE thanks to the fluorophobic alkyl chains and the reduced *Ω*. The entropic change of C6I6 mixed with FE (Δ*S*_mix_, related to fluorophobicity)^22^ and *Ω* of C6I6 have the similar level as those for C6I6 mixed with water. And the low entropy of C6I6 in FE determines its low diffusion in FE (Fig. 1f, Supplementary Note). Besides low diffusion, the second strategy for the stable distribution is low surface tension of ionic liquids. Because of the structure with charge groups encapsulated by alkyl chains, C6I6 has a contact angle (64°) smaller than C1I6’s (82°) on the surface of pure FE, indicating that C6I6 has smaller surface tension in FE (Fig. 2c). Owing to the compatibility of these structural design strategies, C6I6 meets the need for an optimal combination of hydrophobicity and stable distribution in FE.

FE-C6I6 composite (the mass ratio of FE to C6I6 is 2:1) is highly stable in seawater (3.6 wt% NaCl solution) with negligible penetration or leakage, making it mechanically and electrically stable. To monitor the stability in seawater, we compare our composite with other conventional ionic liquids. We firstly immersed composites of FE with different ionic liquids in seawater and monitored ionic conductivities using impedance spectrum (Supplementary Note). As shown in Fig. 2d, Our FE-C6I6 composite exhibited nearly no change in resistance (*R*_B_), associated with ionic diffusion, after immersing in seawater for seven days. The increase in capacitance of the electrical double layer (*C*_EDL_) is due to the lack of drying of the composites. Water increases the dielectric constant of a surface when compared to a dry one. However, all composites other than FE-C6I6 showed obvious changes in resistance due to the production of hydrated ions (Fig. 2e, Supplementary Fig. 1). Weight analysis results further verify the minimum leakages in water. The weight of FE-C6I6 remains above 99.9% after one week of seawater immersion and there is no change in shape (Fig. 2f, Supplementary Fig. 2). Combining the two stability tests, the underwater stability of the material is not only related to the hydrophobicity of the ionic liquid but is also influenced by the stability of the composites. In any case, FE-C6I6 has the best stability among the composites studied.

Based on the study of ionic liquids, the next step is to investigate the distribution of ionic liquids in FE. Their distribution is critical to stability because ionic liquids act as a better pathway for seawater penetration than fluorinated polymers. Another reason is that these composites have different stability although they have the same hydrophobic surface as FE (Supplementary Fig. 3). We can visualize the water penetration using laser scanning confocal microscopy (LSCM) images of the samples after immersing in water with fluorescein solutions for one week (Fig. 2g, Supplementary Fig. 4). The other composites including FE-EMITFSI, FE-HMITFSI and FE-C1I6 all exhibited penetration of water. In our FE-C6I6 composite, however, no water penetration was observed and the material itself remained dark under LSCM. Atomic force microscopy (AFM) results showed that FE-C6I6 was very homogeneous and stable even in the nanometer scale (Supplementary Fig. 5). Although the other materials are also very flat, they have different degrees of phase separation. These results are consistent with the LSCM images and suggest that water penetration is more of a problem for inhomogeneous materials. The cross-section scanning electron microscope (SEM) images of the film exhibit that FE-C6I6 is a solid material without any liquids or holes (Supplementary Fig. 6), further proving the high homogeneity. In summary, the ultra-high underwater stability comes from two folds: (i) high hydrophobicity of C6I6 and (ii) stable and homogeneous distribution of C6I6 in FE. The ultra-high stability in water ensures material work in water for a long time without any mechanical or electrical decays.

Our FE-C6I6 composite exhibits good mechanical strength as well as self-healing capabilities in different aqueous environments (Fig. 2h). It exhibits a Young’s modulus of 0.71 MPa, comparable to commercial soft rubbers (Supplementary Fig. 7). The mechanical performance can remain stable in different water environments for 1 week without any decay. With high Young’s modulus, FE-C6I6 can be molded into different microstructures (Fig. 2i, Supplementary Fig. 8). These microstructures can sustain compressive strengths up to 110 MPa without structural damages (Supplementary Fig. 9). These results eliminate the concerns of structural damages in deep sea. Because of the ion-dipole interactions and the plasticizing effect enhancing the diffusion and entanglement of FE polymers^23–25^ (Supplementary Note), our FE-C6I6 also possessed self-healing capabilities in water (Fig. 2h). When we cut the sample into halves and let them heal in water for 12 h, the healed sample can be stretched up to 300% strain (Fig. 2j, Supplementary Fig. 10).

**Fig. 3:**
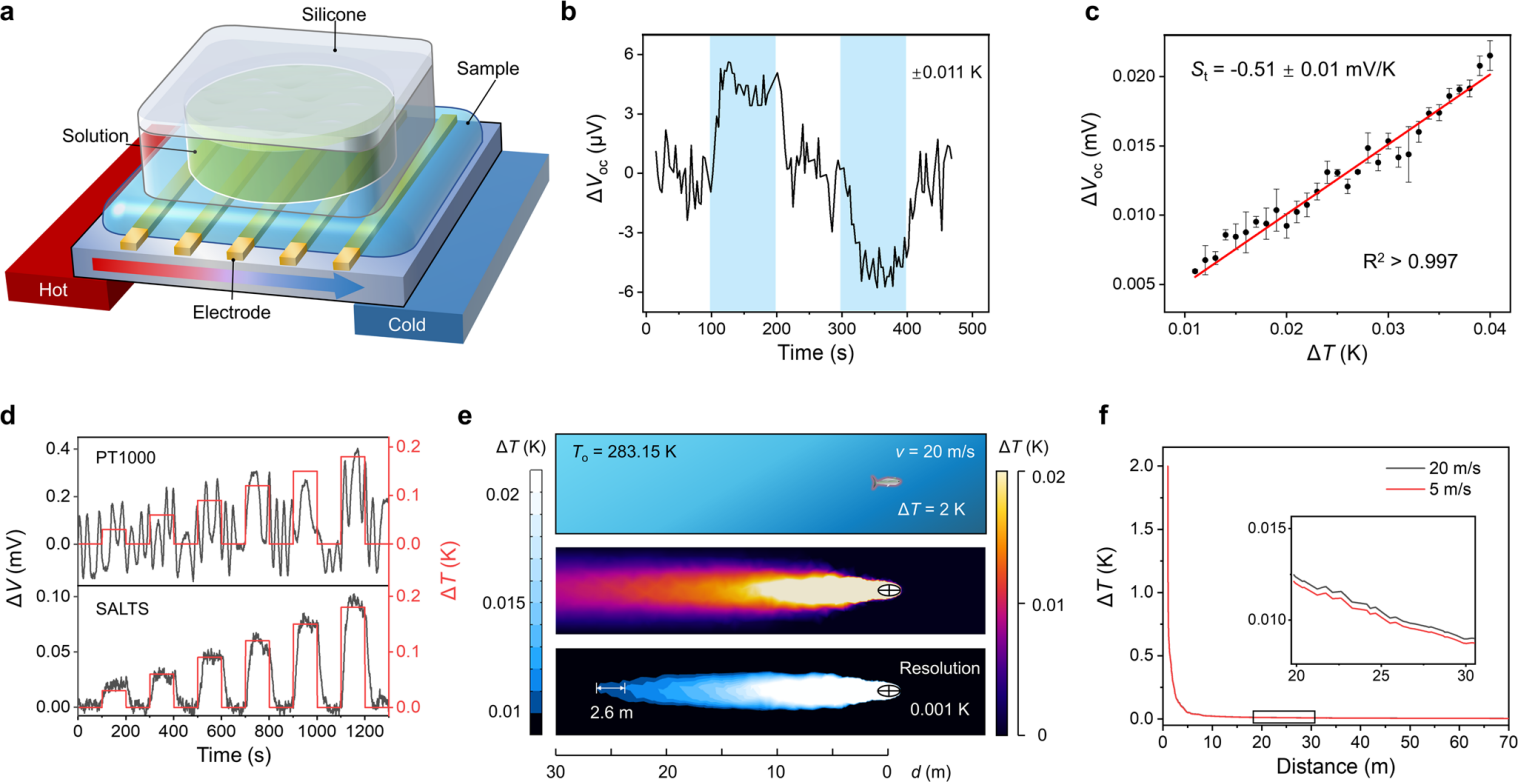
Temperature responses with low response temperature and high resolution. **a** Device for testing the ionic thermoelectric properties of thin film in different water conditions. The arrow represents the direction of the temperature gradient. **b** Time evolution curve of open circuit voltage change (Δ*V*_oc_) in response to Δ*T* = ± 0.011 K with the thin film device. Δ*V*_oc_ is different from *V*(*T*_H_) – *V*(*T*_C_). **c** Dependence of Δ*V*_oc_ on Δ*T*. The resolution is 0.001 K. Error bars show s.d., *n* = 3. **d** Continuous and synchronous time evolution curves of Δ*T* (red curve) and voltage change (Δ*V*, black curve) recorded with PT1000 and SALTS. PT1000 operates at 0.5 mA. **e** Finite element analysis of the heat traces left by fish (Δ*T* = 2 K) swimming (*v* = 20 m/s) in the water (*T*_o_ = 283.15 K). The middle figure shows temperature profile (Δ*T* < 0.3 K) for deep sea environment, around the fish. The bottom image shows the heat trace with the resolution of 0.001 K. **f** Distance evolution curves of temperature on the central axis of the fish (parallel to the direction of swimming) at different swimming speeds. The zero point is at the centre of the fish.

To demonstrate the thermal response of FE-C6I6 in ocean environments, thin films were incorporated into a substrate with parallel electrodes and covered by a silicone container without a bottom (Fig. 3a). The liquid inside the container comes into direct contact with the film underneath. A temperature gradient was applied across electrodes using two Peltier devices. FE-C6I6 has a stable coefficient of −0.51 mV/K. The signals of the composites with other ionic liquids are complex with irregular undulations (Supplementary Note). We measured the responses of FE-C6I6 in a 3.6 wt% NaCl solution over 0.001~0.04 K (Methods). In the range of 0.011 to 0.040 K, a clear linear correlation between voltage and temperature is founded with a resolution of 0.001 K and the minimum value greater than three times the standard deviation of the baseline (Fig. 3b, c). The self-powered nature of the thermoelectric material makes itself free of any voltage or heat in the absence of temperature changes. As a result, the sensor exhibits better real-time temperature detection performance than a metal resistance temperature detector (RTD), which requires power and is self-heating. PT1000, a typical RTD, cannot discriminate temperature variations from 0.03 K to 0.18 K, which can be detected linearly by SALTS (Fig. 3d). To express the value of such a small temperature response, we carried out finite element analysis (FEA) of the heat traces left by fish swimming in the water. Fig 3e shows that the trace of 0.01 K above ambient temperature can reach 26 meters. With a resolution of 0.001 K, a detection range of 2.6 m is sufficient to detect the direction of the temperature gradient. Meanwhile, increasing the resolution value increases the detection range exponentially and reduces its practicality (Supplementary Fig. 11). And its distance is insensitive to the speed of movement (Fig. 3f). The traces left by hotter objects will also be longer. Such distance is substantial to track fish or other moving objects.

**Fig. 4:**
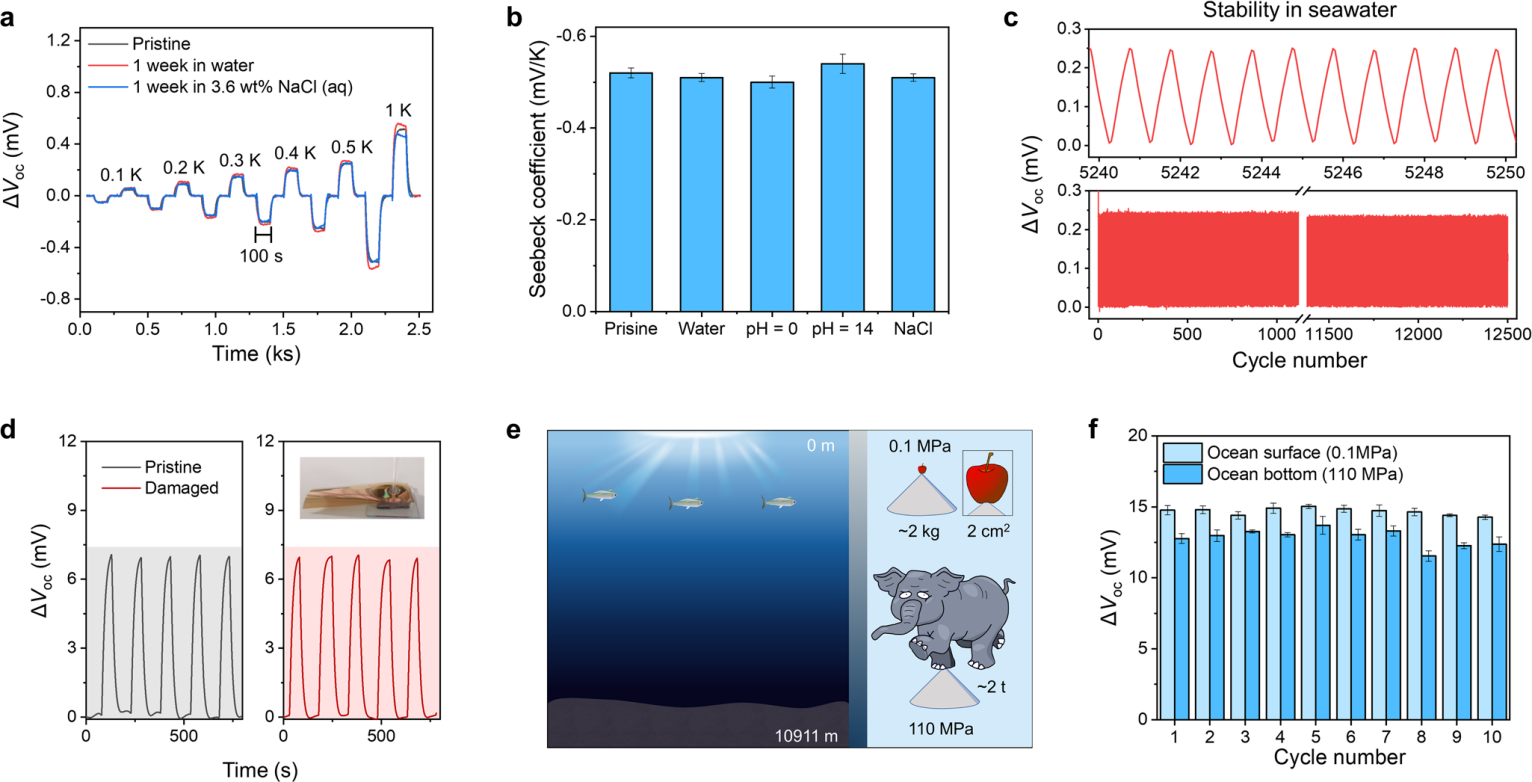
Thermoelectric stability to cope with the harsh environment of the deep sea. **a** Thermoelectric signals of the thin film device after prolonged underwater immersion. **b** Seebeck coefficients of thin films in different water conditions. Error bars show s.d., *n* = 3. **c** Stability of Δ*V*_oc_ in response to the same temperature change for 12,500 cycles. **d** Thermoelectric signals of SALTS before and after being damaged. The inset shows SALTS punctured by the sharp object. **e** The relationship between depth and pressure in the sea. The right image shows the difference between 0.1 MPa and 110 MPa by cartoons. **f** Stability of Δ*V*_oc_ under 0.1 MPa and 110 MPa for 10 cycles. Error bars show s.d., *n* = 3.

To ensure the reliability of such small value for the operations, the long-time stability in the deep sea is important. Fig 4a exhibits the identical thermal voltage changes of pristine FE-C6I6 with that of the same device submerged for 1 week in water or a 3.6 wt% NaCl solution. Cycling tests have shown the sensor’s impressively stability at 12,500 cycles (Fig. 4b). Likewise, the sensor has the same adaptability and stability in different conditions as the FE-C6I6 itself. The nearly identical voltage curves are observed in samples in water, acid, alkaline and saline solutions, indicating stability (Fig. 4c). The thermoelectric property is present in the common seawater temperature range of −10 to 50 °C (Supplementary Fig. 12). Even in a damaged condition, the sensor works and maintains the same temperature responsiveness (Fig. 4d). The enormous pressure of the deep sea is the final hurdle affecting stability. With the use of an oil pressure supply unit (Supplementary Fig. 13), the pressure-insensitive responses are shown in Fig. 4f. The normalized responses are almost close to 1 whether at 0.1 or 110 MPa, which is consistent with deep sea pressure. The stability after repeated pressure cycles is still present. Combined with the fact that the solubility of non-volatile liquids is not pressure sensitive, these data ensure that SALTS can be used in the deep sea, even after multiple deep dive missions.

The excellent stability exhibited by the thermoelectric properties comes from the stability of the ion movement. Based on the Onsager transport theory^8,13,26^ (Supplementary Note), the Seebeck coefficient of symmetrical electrolyte without reactions can be derived as ${S}_{{{{{{\rm{t}}}}}}}=\frac{{D}_{+}{\hat{S}}_{+}-{D}_{-}{\hat{S}}_{-}}{e({D}_{+}+{D}_{-})}$. D is the mass diffusion coefficient, $\hat{S}$ is the Eastman entropy of transfer, e is the elementary charge, and “+” or “−” represent cations and anions, separately. $\hat{S}$ is essentially related to the interaction between ions and the surrounding polymers^27^. The hydrophobicity and dynamics of the interactions endow the coefficient with underwater stability and pressure insensitivity. Meanwhile, $D\hat{S}/{k}_{B}T$ (${k}_{B}$ is Boltzmann constant) is thermal mobility. Therefore, the Seebeck coefficient is determined by the difference in thermal mobilities of the cations and anions. The negative coefficient suggests that TFSI^−^ has the larger thermal mobility. The hydrophobic and fluorophilic natures of TFSI^−^ ensure its stable mobility in FE without interference from water molecules^28^.

**Fig. 5:**
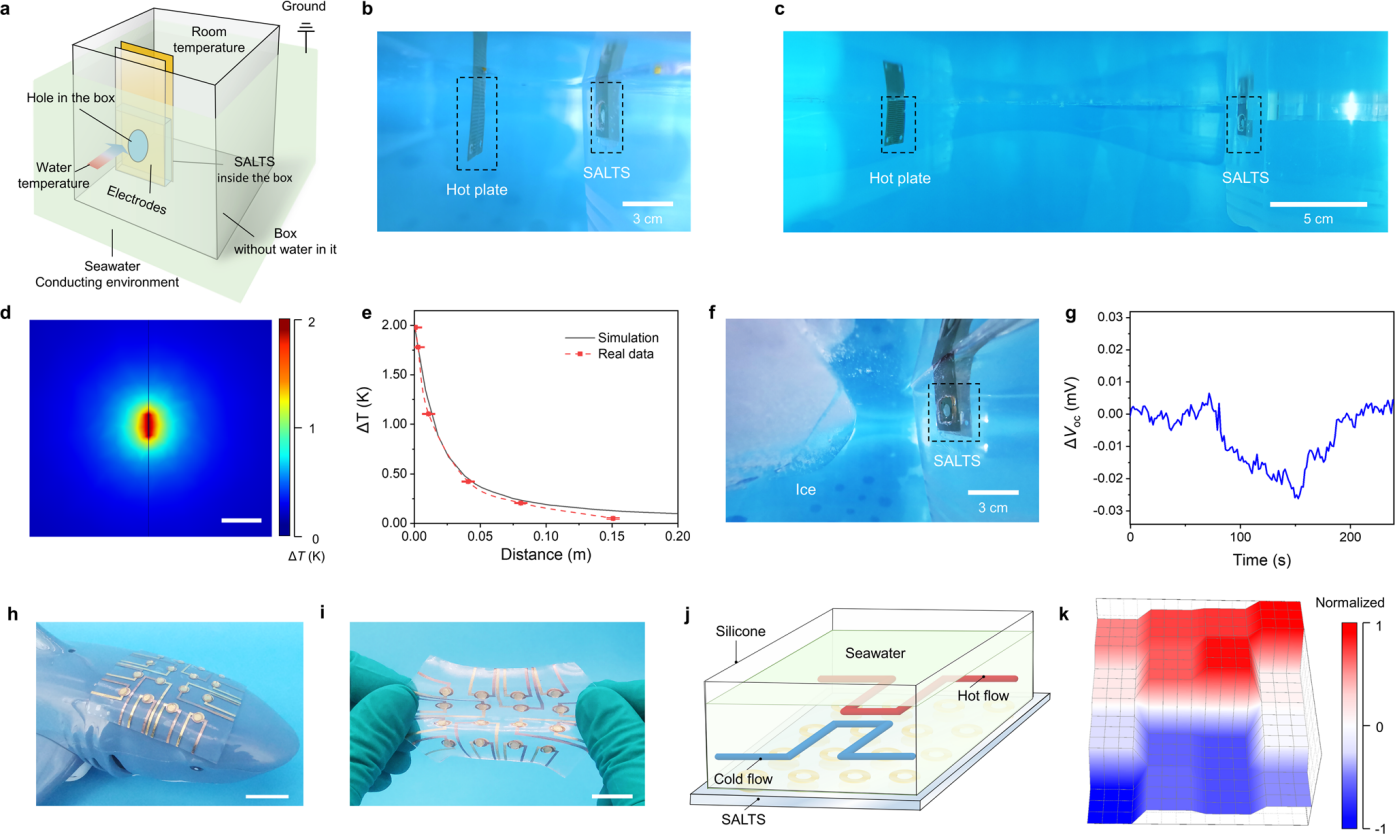
SALTS for temperature sensing and imaging of distant objects underwater. **a** Structure of the underwater temperature sensing system with SALTS. Grounded water simulates a vast ocean. **b**, **c** SALTS sensing a hot plate at different distances. **d** Temperature profile of the water around the hot plate (Δ*T* = 2 K). Ambient temperature, 20 °C. Scale bars, 5 cm. **e** Distance evolution curves of simulated and measured temperature between SALTS and the hot plate. The zero point is at the centre of the hot plate. Error bars show s.d., *n* = 3. **f**, **g** SALTS sensing the emergence of ice. The curve changes in the opposite direction to the heating. **h**, **i** Artificial AoL-like skin with 4-by-4 sensor arrays. Scale bars, 1 cm. **j** SALTS sensing the Π-shaped pipes with hot and cold water flowing through them. Hot water, 30 °C. Cold water, 10 °C. Ambient temperature, 20 °C. **k** Resulting voltage mapping of the array sensing the direction and temperature of the flows.

To mimic the shark nose, we designed a single sensor device shown in Fig. 5a. Typically in deep sea, the temperature differences between seawater and living creatures are around 2 K^29^. Therefore, we generate a 2 K difference with room temperature using a hot plate. We collected the voltage data at different distances away from the thermal sources (Fig. 5b, c). We also use FEA to simulate the distance evolution curves of temperature away from the hot plate (Fig. 5d). The results are highly similar with our experimental results (Fig. 5e). With the detection of temperature gradients, it is possible to deduce the approximate distance and position of the object. Similarly, cold ice could be detected using our single unit device (Fig. 5f, g).

To further use our device to detect and map the object, we fabricated the artificial AoL-like skin with 4-by-4 sensor arrays (Fig. 5h, i). The FE-C6I6 film was sandwiched between patterned electrodes^30^. We emulated the temperature behavior of the heat flow by injecting hot water into the seawater pool above the skin. The position of the heat source was determined by the 3D temperature profile in the skin (Supplementary Fig. 14). To simulate currents in the ocean, hot and cold currents are applied to the skin through Π-shaped copper water pipes. The direction and shape of the currents are clearly revealed by the 3D profile (Fig. 5j, k). Because thermal diffusion makes the temperature at the inlet of the water flow hotter or colder than the temperature at the outlet. Using this SALTS device, we are also able to collect information of water flows and gradients.

In summary, inspired by shark nose, we designed and developed a self-healing, aquatic-stable and ultrasensitive thermal sensor for deep sea exploration. The device can identify a temperature difference as low as 0.01 K with a resolution of 0.001 K. The sensor can work stably in deep sea under a pressure of 110 MPa without any encapsulations. The ultra-low energy consumption and self-powering merits of ionic thermoelectric device can further endow the device with long-term working potential underwater. Our findings can greatly expand human’s techniques for deep sea explorations. And the material’s excellent environment-adaptability promises to broaden the application scenario for electronic skin^31^ and electrochemical energy storage^32^, which are based on ionic conductors.

## Methods

### Preparation of composite solution

Composite solutions were prepared by dissolving FE and ionic liquids (anhydrous) in acetone under stirring for 3~4 h at room temperature. The mass ratio of FE to acetone is 1 (1 g): 7 (9 mL). In the main text, the mass ratio of FE to ionic liquids is 2: 1. For homogeneous mixing, the volume of solution prepared is related to the amount required, for example 1 mL for the preparation of the thin film.

### Preparation of water solutions

The seawater in the experiment parts refers to a NaCl solution. The concentration of the NaCl solution is 3.6 wt%, which is comparable to the salt concentration of seawater. To adapt to the different pH conditions in seawater, 1 mol/L NaOH (pH = 14) and 1 mol/L HCl (pH = 0) solutions were prepared. The solutions were prepared with deionized water.

### Fabrication of FE-C6I6

For 1 to 2 mm thick samples, 8 mL composite solution was poured onto a glass plate (5 × 5 cm^2^) and evaporated naturally for 8 h. After that, the glass plate was further dried at 70 °C in vacuum for 12 h. The film of FE-C6I6 can be easily removed. An appropriate amount of film was taken into the mold for hot pressing (10 MPa, 70 °C, 12 h) by tabletop powder compactor (FY-15, SCJS). Without special mention, the thick samples are all prepared by this method.

For thin samples, 30 μL composite solution was poured onto glass or silicon substrates and evaporated naturally for 8 h. Afterwards, the substrate and the sample were further dried at 70 °C in vacuum for more than 3 h. The film was cooled to room temperature in air. The surface is characterized by this method of fabrication.

Other composites were prepared with the same method. During preparation, the air humidity was 20%, and the room temperature was 25 °C.

### Water solubility measurement

10 mL of ionic liquids was poured into 10 mL of water under stirring for ten minutes at different temperature. After mixing well and waiting for separation of ionic liquids and water, the conductivity of the water was measured by using a conductivity meter (DDS-307A, Rex) with temperature compensation. At constant concentration, the conductivity of the solution increases with increasing temperature. Temperature compensation, which can approximate to eliminate this effect, is the function of the instrument. The coefficient between mass fraction and conductivity is 640 ppm/(mS/cm).

### Impedance measurement

Impedance spectroscopy was measured with electrochemical workstation (CHI660E, CH instruments). The applied AC potential was 50 mV and the frequency scanned from 1 Hz to 1 MHz. The sample was sandwiched with two stainless-steel sheets. Both the sample and the steel sheet have a diameter of 12 mm. The thickness of the sample is 1 mm. Entire sandwich structure was immersed in salt water for 1 week. After that, excess surface water was wiped and stainless-steel sheets were connected to electrodes for testing.

### Water content measurement

We measured the water content of the composites by comparing their weights before and after immersion in 3.6 wt% NaCl solution. After immersion, the composite is rinsed three times with deionized water to remove salt and ionic liquids from the surface. Excess surface water was wiped. The weight before (*m*_*0*_) and after (*m*) immersion was measured. The weight loss was obtained as [(*m*_*0*_ − *m*)/*m*_*0*_] × 100%.

### Confocal characterization

Confocal microscopy was carried out using an Olympus FV3000RS confocal microscope. 2 mL composite solution was poured onto a glass slide (24 × 40 mm2) and evaporated naturally for 8 h. After that, the glass plate was further dried at 70 °C in vacuum for 8 h. After cooling for one to two hours, the sample was immersed in 0.1 wt% fluorescein solution for a week. After immersion, the composite is rinsed with small amount of water deionized water to eliminate the interference of background light. Excess surface water was wiped. The 488-nm laser channel was used to excite the fluorescent water. The two brightest surfaces in the microscope are the upper and lower surfaces. Fluorescent images were collected between the upper and lower surfaces (10 μm).

### Surface characterization

The contact angle data of droplets (2 μL) were collected by a drop shape analyzer (Dataphysics OCA15Pro) at ambient temperature. AFM images were recorded on an Oxford Cypher VRS. SEM images were monitored using a HITACHI SU-8010 SEM device.

### Mechanical testing

Tensile tests were performed using a Sunstest UTM2502. Unless otherwise noted, tensile experiments were performed at room temperature (25 °C) at a strain rate of 10 mm/min for both stretching and relaxing rate. Thick FE-C6I6 were cut into dog-bone-shaped specimens with a width of 4 mm for regular tensile testing. The thickness of the sample was typically around 2 mm. The stress–strain curves were obtained by dividing the measured force by the initial cross-section area and dividing the measured displacement by the initial clamp distance (5 mm). Three samples were tested for each condition.

To verify the underwater self-healing capability, the samples were put into a Petri dish filled with different solutions, and cut into halves with a fresh razor blade. Then, the cut surfaces were gently placed together underwater, and subsequently left underwater at room temperature to permit healing. Tensile tests took place after the designated healing time. The self-healing efficiency was obtained by dividing the self-healed toughness by the initial toughness. Details can be found in previous articles^24,25^.

Press tests were performed using an INSTRON 68TM-5 universal testing machine. Experiments were performed at room temperature (25 °C) at a strain rate of 10 mm/min for both pressing and relaxing rate. Dynamic thermomechanical analysis (DMA) was conducted by TA Instruments DMA 850 at a heating speed of 5 °C min^−1^ from −50 °C to 50 °C.

### Device fabrication

For the thermal voltage measurement of FE-C6I6 film, a glass substrate (1 × 1 cm^2^) with parallel-structured electrodes is utilized to fabricate the measurement device. Gold/chromium (28 nm/2 nm thickness) electrodes with width and length of 10 mm and 500 μm are patterned. 30 μL of composite solution is dropped on cleaned substrate and dried in air for 8 h. After that, the device is further dried at 70 °C in vacuum for 3 h. Finally, a PDMS reservoir (height of 3 mm and diameter of 2.5 mm) is attached on top of FE-C6I6 for liquid holding.

For fabrication of vertical-structured device, a cleaned ITO glass (2 × 2 cm^2^) and gold/chromium (28 nm/2 nm thickness) deposited polyimide film are adopted as the bottom and top electrodes. Pre-fabricated FE-C6I6 (L/W/H: 1.5 cm/1.5 cm/0.3 cm) is sandwiched between two electrodes and dried at 60 °C in vacuum for 3 h.

For measurement in liquid, a hole with diameter of 2.5 mm is punched in top electrode before device fabrication.

The fabrication process of array chip is similar with the vertical-structured device, except that both the top and bottom electrodes are gold/chromium (28 nm/2 nm thickness) deposited polyimide films. 16 devices are arrayed within 4 × 4 cm^2^ and each device’s electrodes are independent for measurement.

### Device measurements

The ionic Seebeck coefficient is calculated by *S*_t_ = ∆*V*/∆*T*, where ∆*V* is the thermal voltage between two electrodes under temperature difference of ∆*T*. The ∆*V* is measured by Keysight B1500A. ∆*T* is introduced by two Peltier elements for parallel-structured electrodes and calibrated as previously reported^33^. The temperature is read from the operating curve of Peltier elements. For vertical-structured device, ∆*T* is introduced by single Peltier element below the bottom electrode and calibrated by detecting the temperature on top and bottom side of FE-C6I6, respectively (Supplementary Note). Different kinds of liquid were added in PDMS reservoir before each measurement. In application of temperature sensing under water, ∆*T* is introduced by electric heating wire or hot/cold copper pipes. For measurement under high pressure, a pressure cell model from Physical Property Measurement System (PPMS, Quantum Design) is used for pressure control and voltage measurement.

### FEA details for heat traces of moving fish and stationary heating plates

The FEA results in this paper were calculated using COMSOL. To validate the heat traces of moving fish (Fig. 3e, f), we built three-dimensional (3D) models of fish (ellipsoid),

heat plates (cuboid) and water flows. Parameters in the FEA are listed in Supplementary Note. The main mode of heat transfer in water is thermal convection. The heat transfer module of COMSOL was used in the simulation. All simulations are based on steady state studies.

## Supplementary information

Supplementary Information

## Data Availability

The data that support the plots within this paper and other finding of this study are available from the corresponding author upon request.

## Code availability

Finite element analysis was performed using COMSOL.
