# Supplementary material for: Solid state ionics enabled ultra-sensitive detection of thermal trace with 0.001K resolution in deep sea
Source: Nat Commun. 2023 Jan 12;14:170. doi: 10.1038/s41467-022-35682-8 (PMC9837202; doi:10.1038/s41467-022-35682-8)
Supplement: Supplementary file 1 — Supplementary Information [file 41467_2022_35682_MOESM1_ESM.pdf]

Supplementary Information for

Solid state ionics enabled ultra-sensitive detection of thermal trace with 0.001 K resolution in deep sea

Yucheng Zhang[1,*], Dekai Ye[2,3,*], Mengxue Li[1], Xi Zhang[1], Chong-an Di[2,†], Chao Wang[1,†]

[1]*Key Lab of Organic Optoelectronics & Molecular Engineering, Department of Chemistry, Tsinghua University, Beijing 100084, China.*

[2]*Beijing National Laboratory for Molecular Sciences, CAS Key Laboratory of Organic Solids, Institute of Chemistry, Chinese Academy of Sciences, Beijing 100190, China.*

[3] *Zhangjiang Laboratory, 100 Haike Road, Shanghai 201210, China.*

*These authors contributed equally to this work.

[†]Correspondence to: chaowangthu@mail.tsinghua.edu.cn (C.W.); dicha@iccas.ac.cn (C. D.).

Table of Contents

Supplementary Figures

Materials

Supplementary Methods

Synthetic Procedures

Supplementary Note

1. Entropy in relation to the structure of cations
2. Entropy and surface energy in relation to the stable distribution
3. Electrochemical impedance spectroscopy for underwater stability (surface and body)
4. Seebeck coefficient based on Onsager theory
5. Interactions between ionic liquids and FE polymers
6. Mechanical properties affected by ionic liquids
7. Ionic thermoelectric properties affected by ionic liquids
8. Parameters for finite element analysis (FEA)
9. Calibration of measured temperatures
10. Temperature resolution in saturated solution of NaCl
11. Thermal conductivity of FE-C6I6

Supplementary Reference

Supplementary Figures

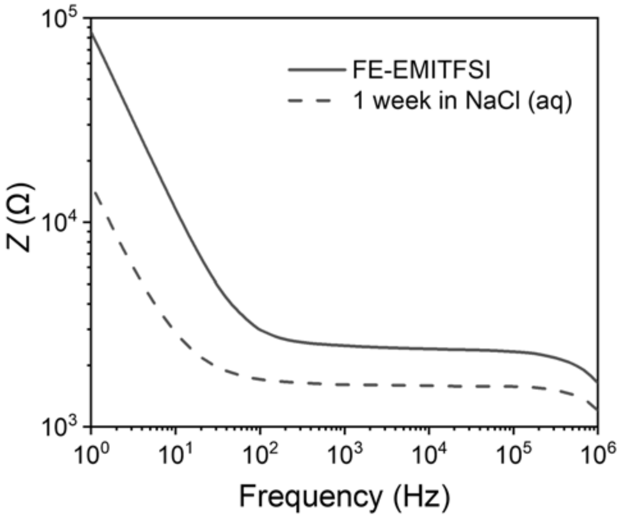

Supplementary Fig. 1 | Bode plots obtained from the original FE-EMITFSI sample and the sample immersed in 3.6 wt% NaCl (aq) for a week.

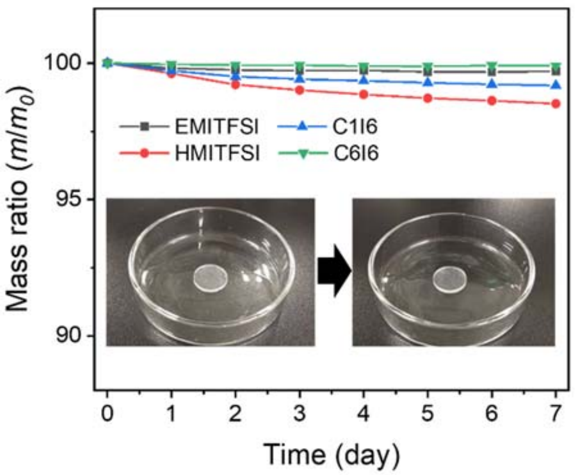

Supplementary Fig. 2 | Time evolution curves of mass ratio of the material before (m_0) and after immersion (m) in 3.6 wt% NaCl (aq). The inset shows round cake shaped FE-C6I6 before and after a week's immersion. The mass ratio of FE to ionic liquids is 2: 1.

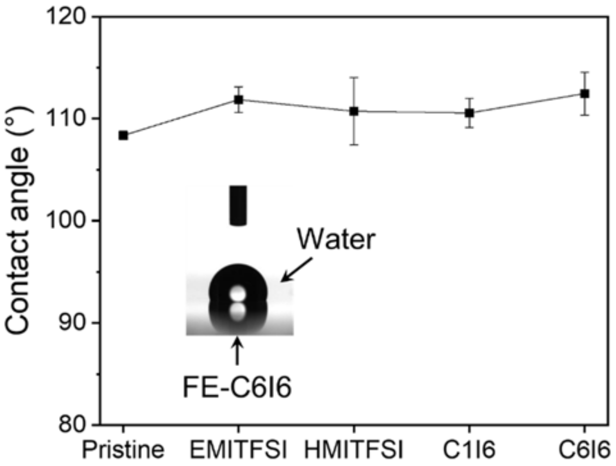

Supplementary Fig. 3 | Contact angle test with an water droplet (2 μL) on bare FE-XX. The mass ratio of FE and XX is 2: 1. XX is an ionic liquid. Error bars show s.d., n = 3.

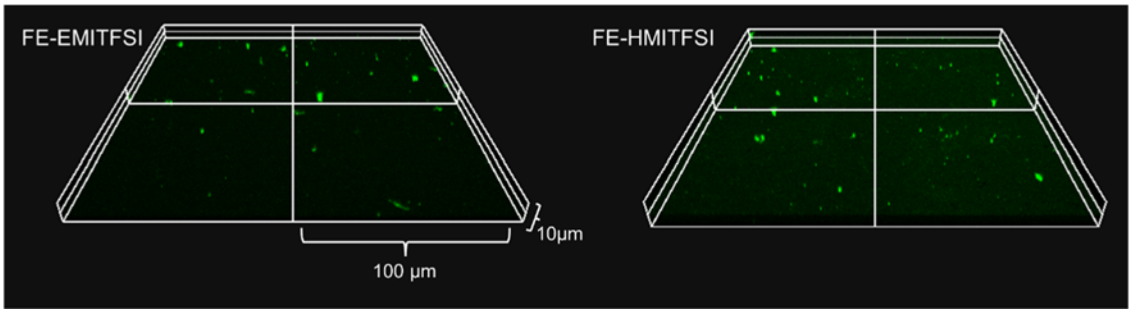

Supplementary Fig. 4 | LSCM images of the sample immersed in a 10 mmol fluorescein solution for a week. Magnification, 60x. The mass ratio of FE to ionic liquids is 2: 1.

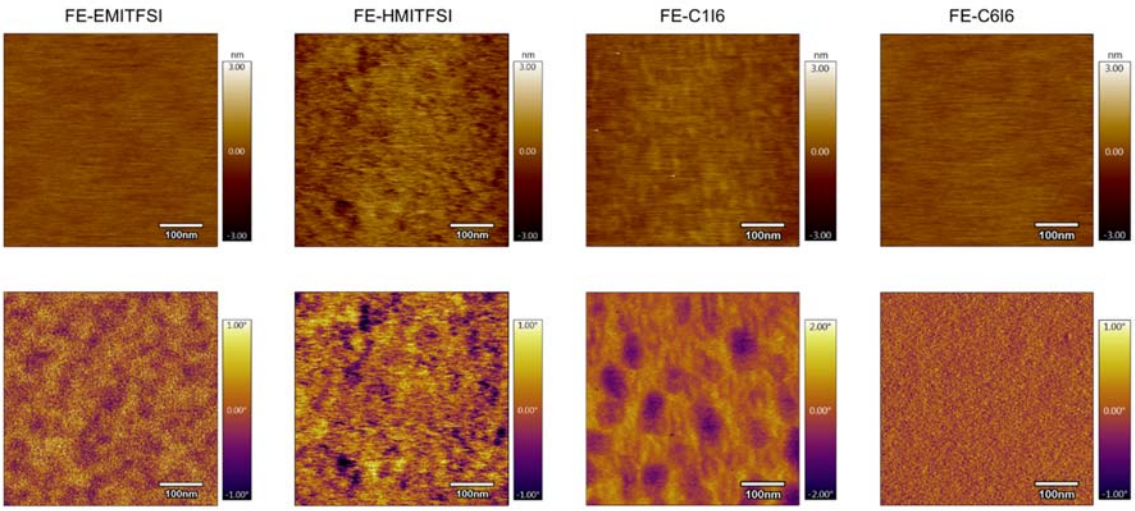

Supplementary Fig. 5 | AFM images of the composites film. The top line shows topography images. The bottom line shows phase images. Bright yellow and orange regions in the phase images correspond to the rigid parts containing less ionic liquids. And dark brown and black regions correspond to the soft parts containing more ionic liquids. The mass ratio of FE to ionic liquids is 2: 1. Scale bars, 100 nm.

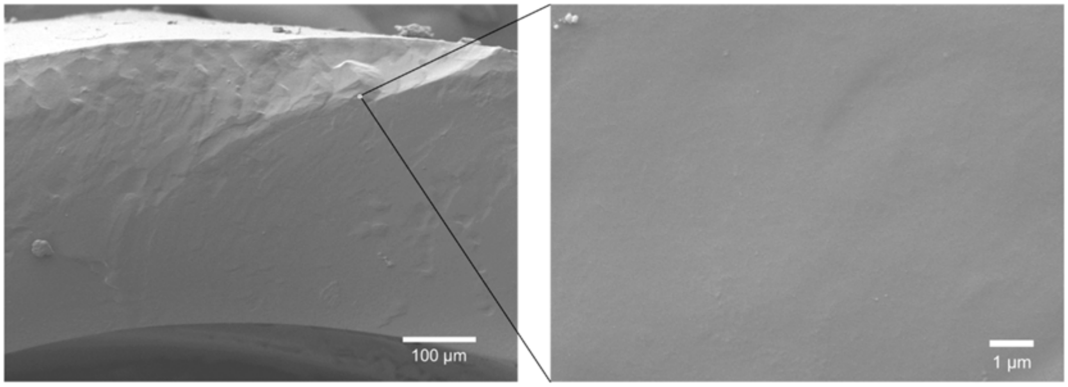

Supplementary Fig. 6 | Cross-section SEM images of FE-C6I6.

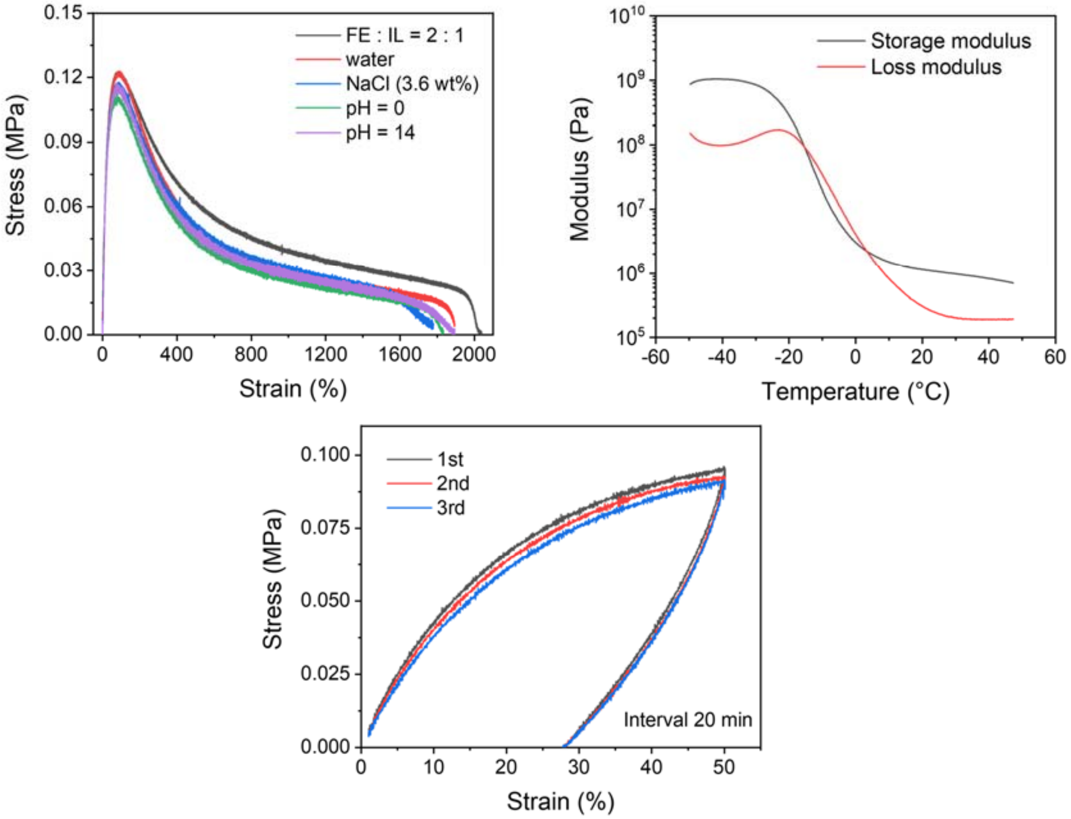

Supplementary Fig. 7 | Elasticity of FE-C6I6. Left: stress–strain curves of FE-C6I6 after 1 week of immersion in different water solutions. Right: dynamic thermomechanical analysis (DMA) of FE-C6I6. Bottom: cyclic loading of FE-C6I6 to 50% strain.

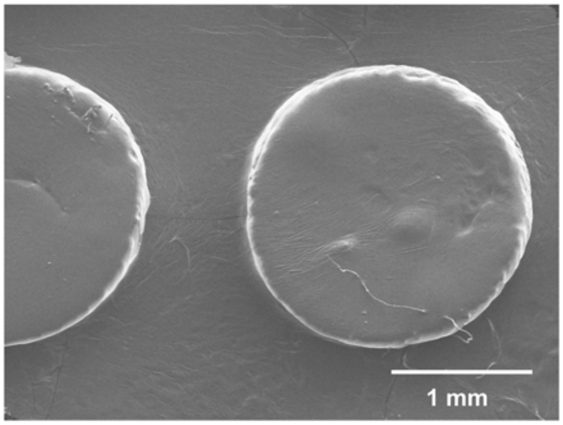

Supplementary Fig. 8 | SEM image of FE-C6I6 with an array of micropillars on the surface.

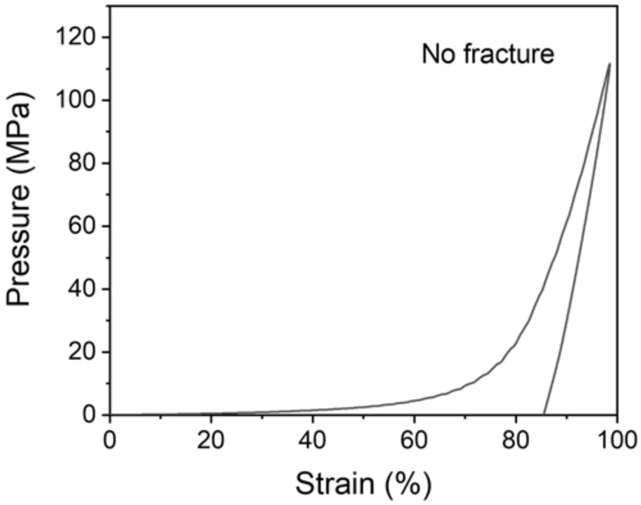

Supplementary Fig. 9 | Pressure–strain curve of column shaped FE-C6I6. Diameter, 7.2 mm. Height, 2 mm. Strain rate, 10 mm/min.

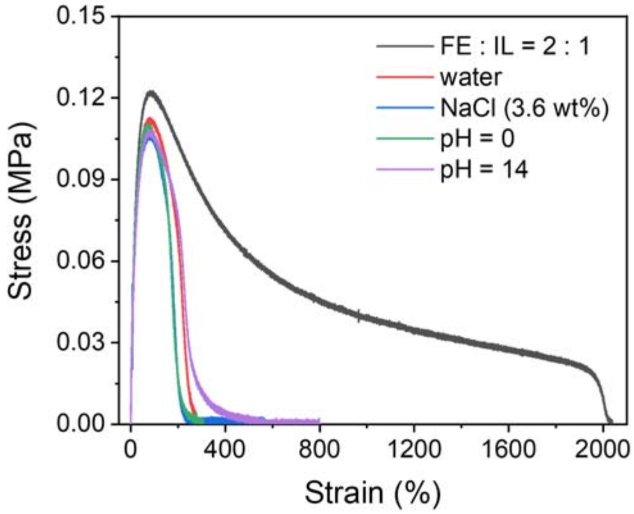

Supplementary Fig. 10 | Stress–strain curves of FE-C6I6 after 12 h of self-healing in different water solutions.

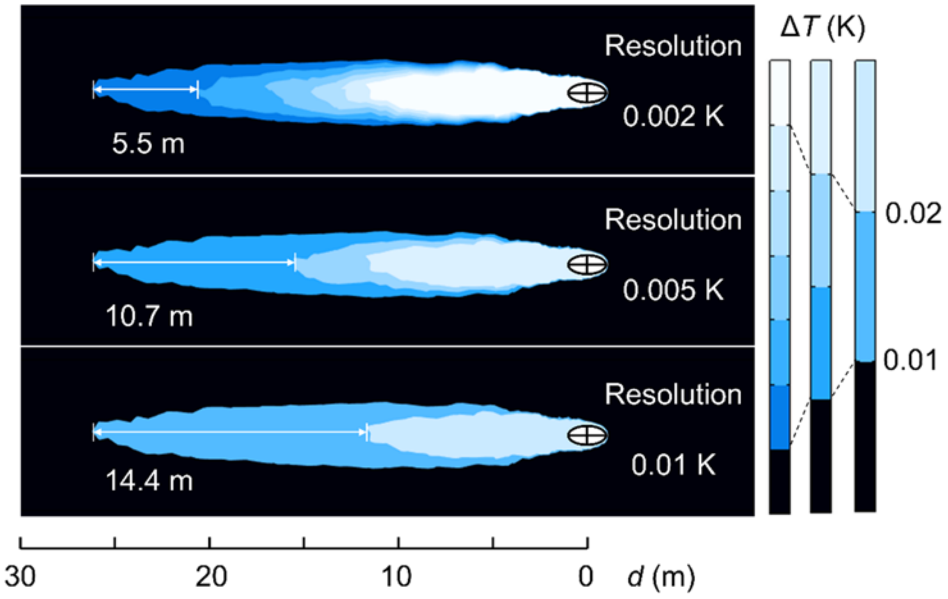

Supplementary Fig. 11 | FEA of heat traces with different resolutions.

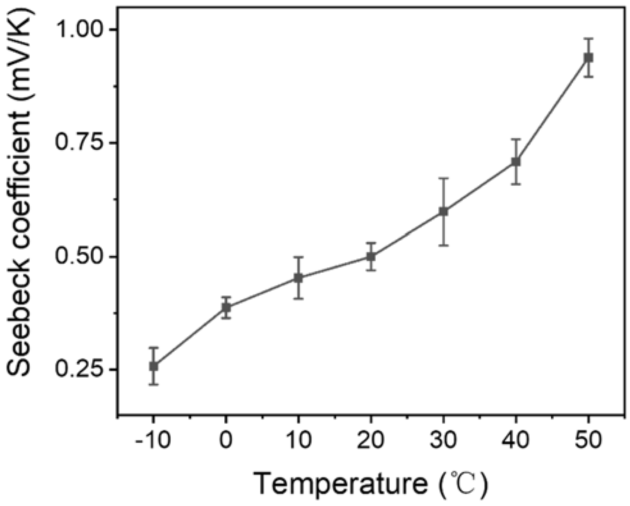

Supplementary Fig. 12 | Seebeck coefficients in the range of -10 to 50 °C. Error bars show s.d., n = 3.

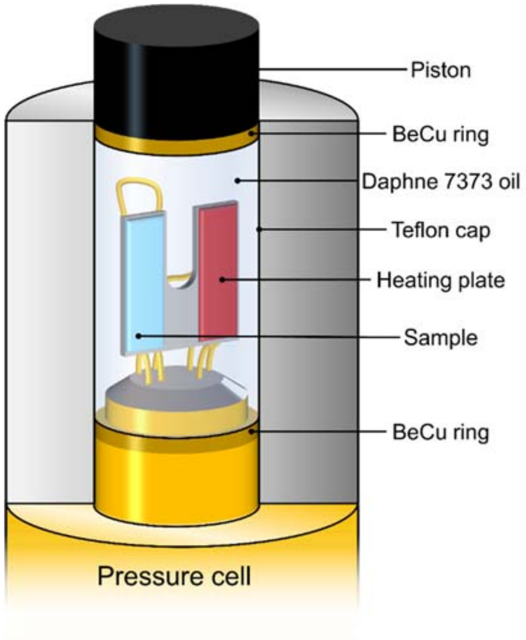

Supplementary Fig. 13 | Structural diagram of the pressure cell. The oil is pushed by the piston to create a very high oil pressure.

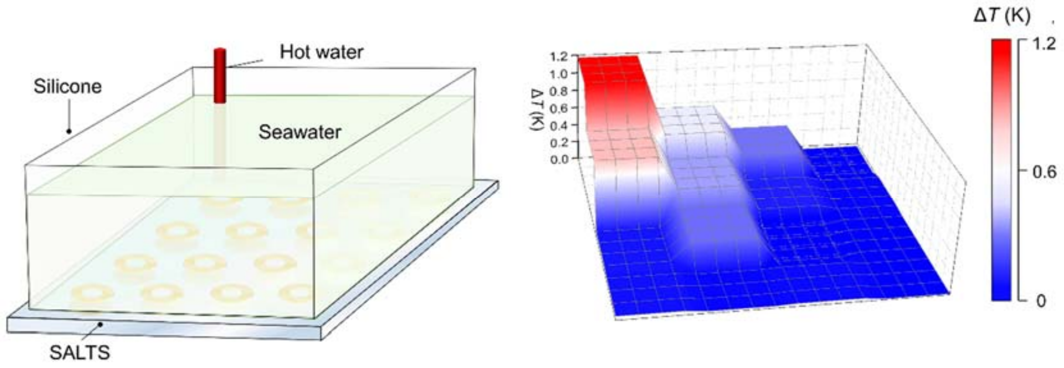

Supplementary Fig. 14 | SALTS sensing the orientation of the heat source. Heat source (2 mL water), 30 °C. Ambient temperature, 20 °C. Right: resulting temperature mapping of the array sensing the orientation.

Materials

Fluoroelastomers or poly (vinylidene fluoride-co-hexafluoropropylene) (G-801) were purchased from DAKIN, and the mass fraction of fluorine is 66%. 1,6-Dibromohexane, N-hexylimidazole, N-methylimidazole, EMITFSI, HMITFSI, and lithium bis(trifluoromethanesulphonyl)imide (LiTFSI) were purchased from TCI. Hydrochloric acid (37%), sodium hydroxide and sodium chloride were purchased from Tong Guang (Beijing). All chemicals were used as purchased without further treatment.

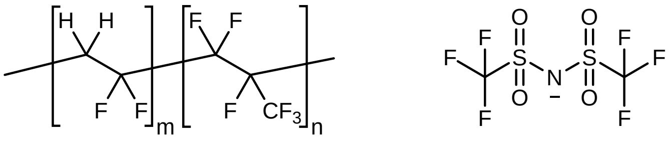

FE TFSI$^-$

Supplementary Fig. 15 | Chemical structures of FE and TFSI$^-$ in the main text. m = 0.55, n = 0.45.

Supplementary Methods

^{1}H NMR (400 MHz) spectra were recorded on a JOEL JNM-ECA400 spectrometer. FTIR data were recorded on a Horiba Bruker FTIR in ATR mode between 400 and 4000 cm^{-1} at room temperature. Differential scanning calorimetry (DSC) tests from −50 to 50 °C with a heating speed of 5 °C min^{-1} were performed using a TA Instruments DSC 250.

Synthetic Procedures

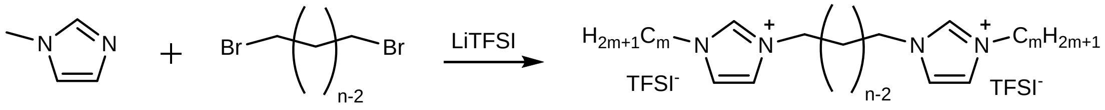

Supplementary Fig. 16 | Synthesis of CmIn.

The synthetic routes for the C6I6 is shown as follows:

1,6-Dibromohexane (4.88 g, 0.02 mol) was added into N-hexylimidazole (12.18 g, 0.08 mol). The reaction mixture was continued stirring overnight at 70 °C. After being cooled to room temperature, the reaction mixture was instilled into water. The aqueous solution was extracted and separated three times with ethyl acetate. Then lithium bis(trifluoromethanesulphonyl)imide (LiTFSI) (17.22 g, 0.06 mol) was added into the water solution. Because C6I6 is hydrophobic. C6I6 can be separated from water. Then the phase of C6I6 was collected and was washed by water for at least three times. Finally, the crude product was further purified in the oven under vacuum at 70 °C. C6I6 is a transparent, yellowish, viscous liquid (17 g, 90%). ^{1}H NMR (400 MHz, Acetone-d_6): δ 9.10 (d, J = 1.7 Hz, 2H), 7.82 (dt, J = 13.1, 1.9 Hz, 4H), 4.39 (q, J = 7.1 Hz, 8H), 2.05 – 1.92 (m, 8H), 1.56 – 1.43 (m, 4H), 1.43 – 1.24 (m, 12H), 0.94 – 0.81 (m, 6H). ^{13}C NMR (101 MHz, Acetone-d_6): δ 135.77, 122.77, 122.71, 121.71, 118.52, 49.73, 49.58, 30.90, 29.79, 29.65, 25.57, 25.40, 22.14, 13.27. HR-ESI-MS (m/z): calcd. for $[C_{24}H_{44}N_4]^{2+}$ 194.18, found 194.18.

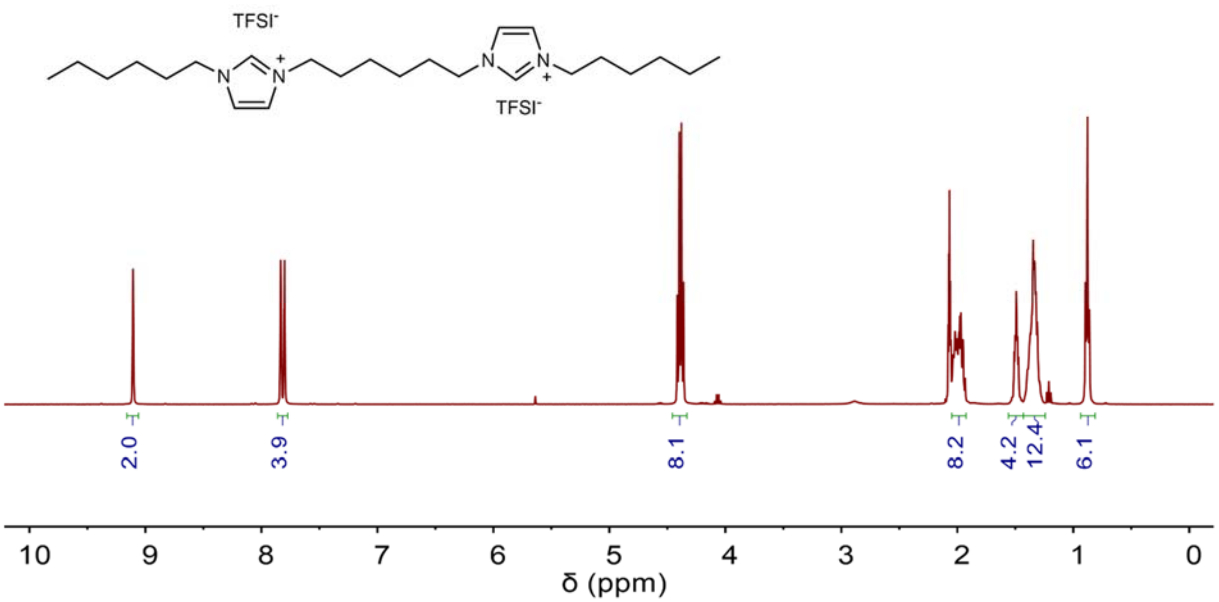

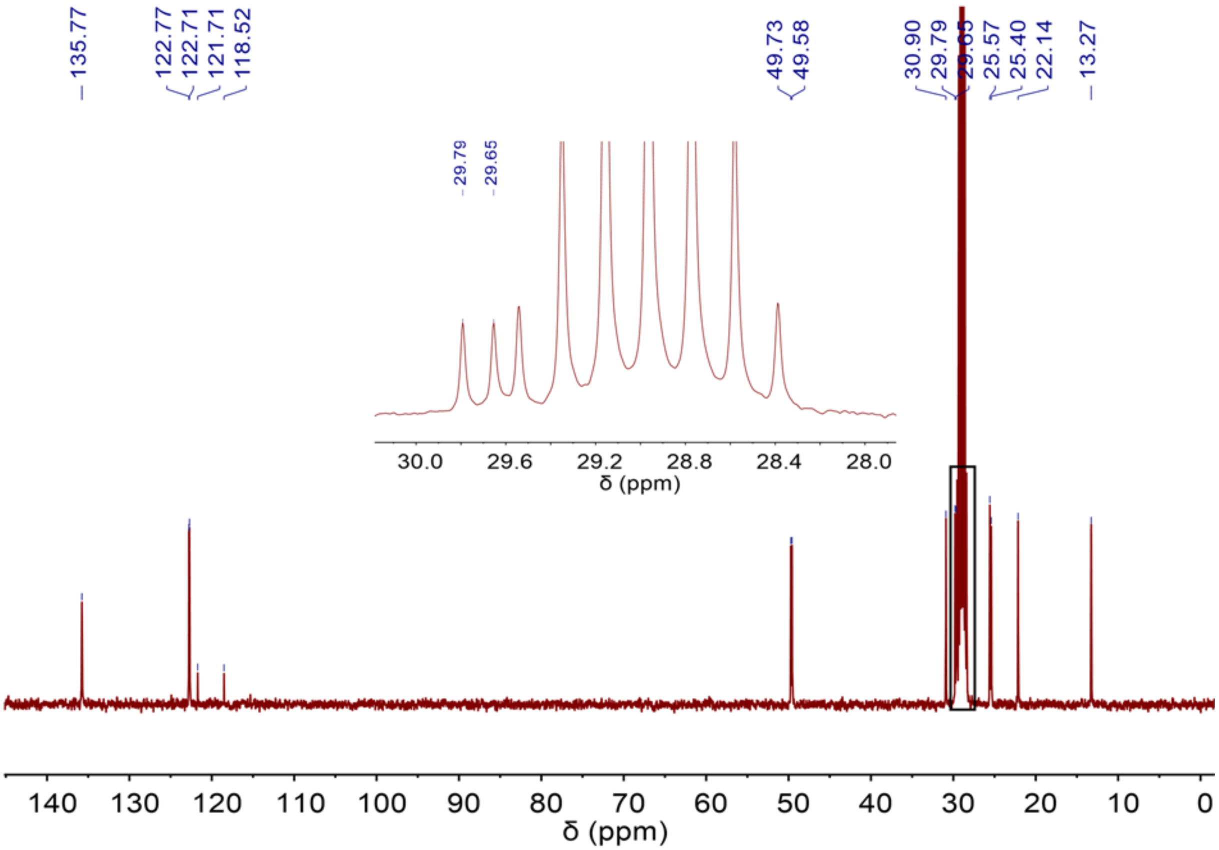

Supplementary Fig. 17 | ^{1}H NMR (400 MHz, Acetone-*d*$_6$, 298K) spectrum of C6I6. ^{13}C NMR (101 MHz, Acetone-*d*$_6$, 298K) spectrum of C6I6.

C1I6 was synthesized with the same procedure and C1I6 is a transparent, yellowish, viscous liquid. ^{1}H NMR (400 MHz, Chloroform-*d*): δ 8.51 (s, 2H), 7.34 (dt, J = 23.1, 1.7 Hz, 4H), 4.12 (t, J = 7.3 Hz, 4H), 3.86 (d, J = 1.3 Hz, 6H), 1.84 (d, J = 7.2 Hz, 4H), 1.35 (d, J = 6.6 Hz, 4H). ^{13}C NMR (101 MHz, $CDCl_3$): δ 135.57, 123.66, 122.38, 121.31, 118.12, 49.56, 35.91, 29.28, 24.89. HR-ESI-MS (m/z): calcd. for $[C_{14}H_{24}N_4]^{2+}$ 124.10, found 124.10.

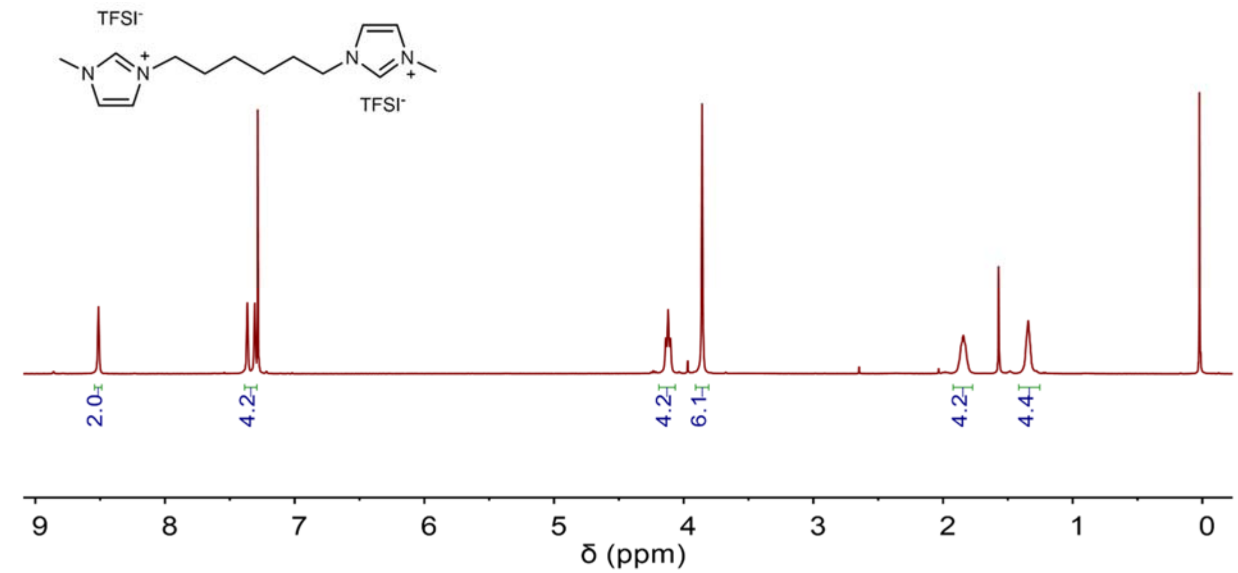

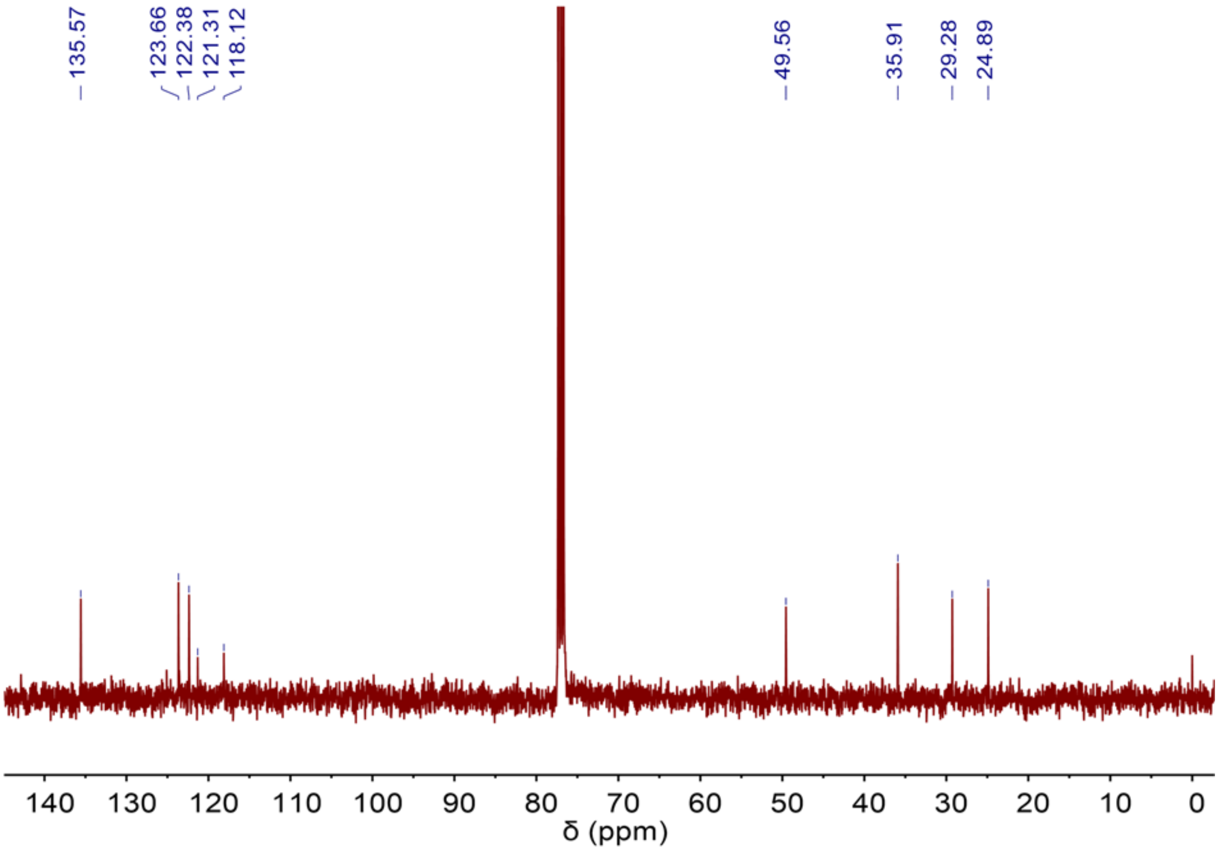

Supplementary Fig. 18 | ^{1}H NMR (400 MHz, Chloroform-*d*, 298K) spectrum of C1I6. ^{13}C NMR (101 MHz, Chloroform-*d*, 298K) spectrum of C1I6.

Supplementary Note

1. Entropy in relation to the structure of cations

Since solubility product (K_{sp}) is related to the concentrations of dissolved ions, we can calculate K_{sp} if we can measure the concentrations of the ions in solution. Meanwihile, in dissolution equilibrium, Equation (1) takes the form of a straight line equation, which provides us with a method to estimate the values of dissolution enthalpy (ΔH_{sol}) and dissolution entropy (ΔS_{sol}). Equation (1) is as follows:

$$\ln\left(K_{sp}\right) = -\frac{\Delta H_{sol}}{RT} + \frac{\Delta S_{sol}}{R} = -\frac{\Delta G_{sol}}{RT} \quad (1)$$

R is the gas constant (8.314 J·K^{-1}·mol^{-1}).

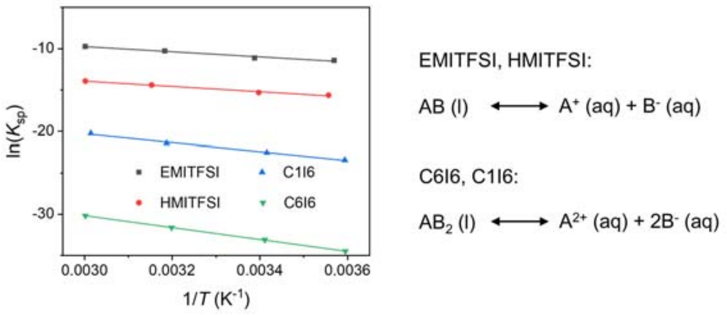

Supplementary Fig. 19 | Linear fitting of solubility product (K_{sp}) to temperature (T). K_{sp} is calculated from the chemical equations on the right of the graph. A, cation. B, anion.

Table 1 shows that the enthalpy and entropy values of EMITFSI and HMITFSI are almost half those of C1I6 and C6I6. This is because the number of charges in the AB-type equation is half that of the AB_2-type equation (Supplementary Fig. 19). To have the same number of charges in the equations, the chemical equations are changed (Left, Supplementary Fig. 20). Since the anions of

the four ionic liquids are identical and the cations are the same imidazolium cations, the enthalpy changes of dissociation and hydration of the ions during dissolution are similar. The effect of enthalpy change on dissolution can be ignored.

Supplementary Table 1. Dissolution entropy and enthalpy obtained from fitting the K_{sp} versus temperature relationship

	ΔS_{sol} (J/(mol·K))	ΔH_{sol} (kJ/mol)
EMITFSI	-13±5	-26±3
HMITFSI	-36±4	-27±2
C1I6	-34±7	-45±3
C6I6	-70±8	-60±1

*The content in parentheses is the mass ratio (FE : XX).

From the intercept of the line, the line of EMITFSI is close to that of C1I6, and the straight line of HMITFSI is close to that of C6I6. So, the four ionic liquids can be divided into two groups (EMITFSI and C1I6, HMITFSI and C6I6). Ionic liquids of the same group have similarities in their dissolution behaviour. Ionic liquids in the different groups have significantly different entropy values. This is because different lengths of hydrophobic alkyl chains dissolve in water with different conformational entropy changes. In summary, the longer the side alkyl chains, the lower the dissolution entropy of the ionic liquid.

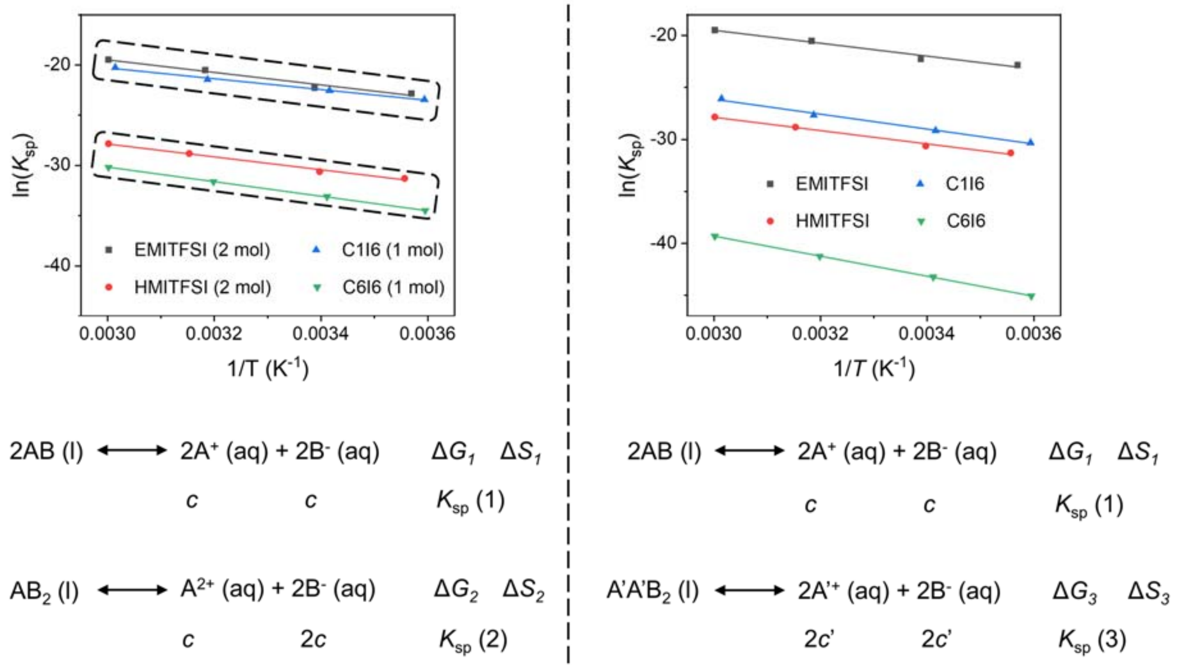

Supplementary Fig. 20 | Linear fitting of solubility product (K_{sp}) to temperature (T). K_{sp} is calculated from the chemical equations below the graph. A, cation. B, anion. c and c' represent solubilities.

Although the above analysis solves some problems, the solubility of ionic liquids in water within the same group still varies greatly (Fig. 2a). This is because the above analysis is based on the dissociation and hydration processes of ions. And the difference between AB and AB_2 is not included. Even though the charge numbers of the equations are the same, the particle numbers are different, which brings about a huge difference between the microstate numbers (Ω). To see the difference in the entropy of the dissolution process due to the microstates, we consider A of AB_2 as two particles (A') (Right, Supplementary Fig. 20).

Based on the law of mass action, K_{sp} (2) = $4c^3$ and K_{sp} (3) = $16c'^4$. Because the value of

solubility $c = c' << 1/4$, K_{sp} (2) $\neq$ K_{sp} (3). As mentioned above “the effect of enthalpy change on dissolution can be ignored”, we can deduce from Equation (1) that $\Delta S_2 \neq \Delta S_3$. So ΔS_3 is not just the dissolution entropy, and also includes a reduction in the number of microstates. Due to the linker of C1I6 or C6I6, A’ is not a truly independent ion, but is in a bound state. Supplementary Fig. 20 shows the difference between A and A’. In summary, the linker can also reduce the entropy of the system, which can reduce the solubility. A detailed thermodynamic derivation of this entropy can be referred to the relationship between bound states and the multivalency effect[1].

At the same time, we can see that treating all ionic liquids as the same particles (AB) best reflects the relationship between entropy and solubility (Fig. 2b, Supplementary Fig. 20). During dissolution, C6I6 has the lowest entropy amongst the ionic liquids mentioned.

2. Entropy and surface energy in relation to the stable distribution

Solid mixtures are different from liquid mixtures because there is no boundary between dissolution and precipitation. So, we only consider compatibility and mixing conditions of components in solid mixtures. The following discussion is based on the fact that ionic liquids and polymers are compatible and homogeneously mixed.

The change in entropy (ΔS), which is related to the dissolution of ionic liquids, also plays an important role in the diffusion of the entire composites. Because dissolution is the process of diffusion of a substance into a solvent, and diffusion process is typically entropically favored. Diffusion (D) of a tagged molecule has a relationship with the entropy (S) of complex systems. The quantitative relation between the two was established by Adam and Gibbs[2,3]:

$$D(T) = A \times \exp\left(-\frac{C}{TS_C}\right) \quad (2)$$

where S_c is the configuration entropy of the system, defined as:

$$S_C(T) = S(T) - S_{Vib}(T) \quad (3)$$

where S_{Vib} is the vibrational entropy of the system. $D(T)$ is the diffusion coefficient at a temperature T. A and C are temperature independent constants. Equation (2) qualitatively and correctly describes the strong dependence of diffusion on entropy. Increased entropy favours diffusion. The entropies discussed in the text all belong to the category of conformational entropy. In our system, it can be understood that increased diffusion leads to ions coming into contact with each other. The resulting aggregates make the material unstable.

The ordering of the entropy of ionic liquids in the composites (ΔS_{mix}) can be analogous to that in water (ΔS_{sol}). Because the side alkyl chain is both hydrophobic and fluorophobic[4], and the bound state of cations with a linker is not related to the environment. In terms of entropy, when C6I6 mixed with polymers, the increase in entropy of the system is minimal among the ionic liquids. The structure of C6I6 still has advantages in entropy (Supplementary Fig. 20, 21).

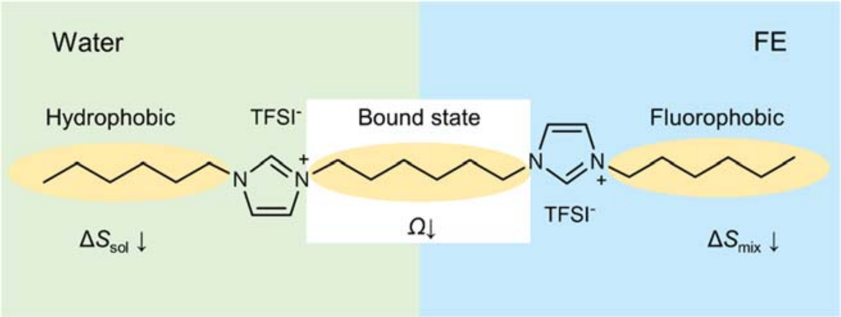

Supplementary Fig. 21 | Relationship between the structure of C6I6 and entropy. ΔS_{sol}, dissolution entropy in water. ΔS_{mix}, entropy of ionic liquids mixed with FE. Ω, the number of the microstates.

Many factors affect the distribution of ionic liquids. One of the major factors is the surface energy of ionic liquids. Surface energy is related to the disruption of intermolecular bonds that occurs when a surface is created. The amount of surface energy determines the size of the stable particles. Small surface energy is beneficial for dispersion into small particles and stable distribution. The contact angle test allows us to compare the surface energy of ionic liquids. As with the preparation process of nanoparticles[5], the introduction of alkyl chains can effectively reduce aggregation of ionic liquids by reducing the surface tension. In terms of surface energy, the structure of C6I6 also has advantages (Fig. 2c).

C6I6 possesses a highly hydrophobic nature and a stable distribution in the composite. This is what makes FE-C6I6 so stable underwater.

3. Electrochemical impedance spectroscopy for underwater stability (surface and body)

Supplementary Fig. 22 show the Bode plot of an ionic conductor. The system does not have redox reaction at the electrode interface. The charge transfer resistance at electrode-electrolyte interface need not to be considered. And electrode resistance is negligible compared to the impedance of the ionic conductor. The equivalent circuit comprises of the capacitance from electrical double layer (C_{EDL}), bulk resistance (ion resistance) (R_B), bulk capacitance (geometrical capacitance) (C_B)[6].

At low frequency (region 1 and 2), the impedance of R_B is much smaller than that of C_B and the parallel part can be approximated as R_B. In region 1, the impedance of C_{EDL} is much greater than R_B, and C_{EDL} is the dominant component of the equivalent circuit. In practice, the impedance of C_{EDL} is not exactly the same as the equation due to interface contact and other problems, and corrections to the equation need to be made. $Z(C_{EDL}) = 1/[(2\pi f)^{\alpha} C_{EDL}]$. α is close to unity. Hence, the slope of the curve in region 1 is – α. The impedance of C_{EDL} decreases with frequency, and R_B becomes dominant in region 2. The plateau line in region 2 corresponds to the frequency-independent nature of R_B. As frequency increases further (region 3), the impedance of C_B decreases and is smaller than R_B. Hence, the parallel part can be approximated as C_B. The impedance of C_B is much greater than that of C_{EDL} at the same frequency, and C_B becomes dominant in region 3.

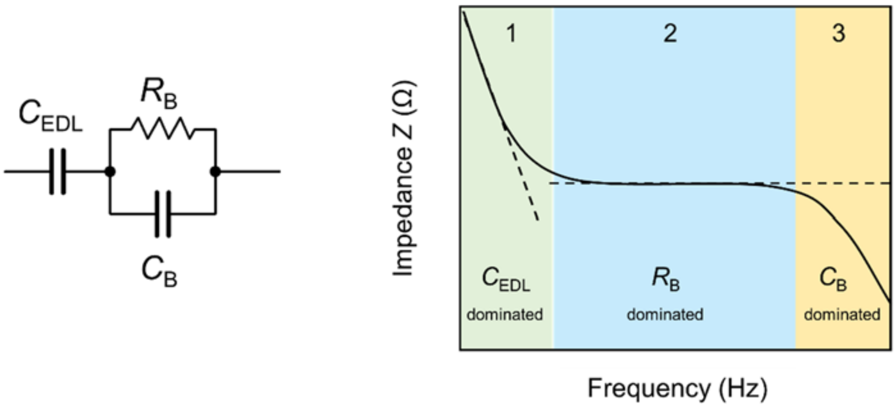

Supplementary Fig. 22 | Equivalent circuit and Bode plot of an ionic conductor.

At region 1, the curve changes can reflect the contact between the surface and the water. Water has a large dielectric constant (78.36 F/m at 25 ℃), about ten times that of polymer materials. Water on the surface significantly increases C_{EDL}. $C_{EDL} \propto \frac{\varepsilon S}{d}$, where ε is the dielectric constant of electrical double layer, S is the area of a activated surface, and d is the thickness of electric double layer[7]. At region 2, the curve reflects whether water in the material affects the movement of ions. The plasticizing effect of water on polymers and the hydration of ions facilitate the movement of ions in polymers.

4. Seebeck coefficient based on Onsager theory

In the study of thermoelectric materials, Onsager theory has been repeatedly verified to be universal[8-10]. All irreversible processes are the result of a generalized thermodynamic flow driven by some generalized thermodynamic force. When there are several generalized forces present at the same time, there are corresponding generalized flows present at the same time and they cross-couple. This means that all the generalized forces (external fields) contribute to each type of flow.

For thermodynamic processes in the linear non-equilibrium region, the relationship between thermodynamic flow (J_p) and force (X_j) can be approximated by the linear phenomenological law, i.e.:

$$J_p = \sum_j L_{pj} X_j \tag{4}$$

L_{pj} or L_{jp} is an Onsager coefficient. and p or j refers to a certain type of flow or force. There are Onsager reciprocal relations, i.e.:

$$L_{pj} = L_{jp} \tag{5}$$

In our system we only consider the heat flow from the temperature gradient and the ion flow from the chemical potential gradient. And the flow of a particular ion can be expressed as:

$$J_i = L_{ii} X_i + L_{iQ} X_Q = L_{ii} \frac{\nabla(-\tilde{\mu}_i)}{T} + L_{iQ} \nabla\left(\frac{1}{T}\right) \tag{6}$$

Among them, Q refers to the heat, and i refers to the ion type.

In the presence of an electrostatic potential V(r), the chemical potential is $\tilde{\mu}_i = \mu_i[n_i(r), T(r)] + q_i V(r)$, where the chemical potential μ_i is a functional of concentration profile $n_i(r)$ and temperature profile $T(r)$. And q_i is the charge of the ion. Therefore, the expression of $\nabla\tilde{\mu}_i$ can be written as:

$$\nabla\tilde{\mu}_i = \left(\frac{\partial \mu_i}{\partial n_i}\right)_T \nabla n_i + \left(\frac{\partial \mu_i}{\partial T}\right)_{n_i} \nabla T + q_i \nabla V \tag{7}$$

And q_i is the number of the ions. The subscript indicates that the variable remains constant when the partial derivative is taken. In an ideal solution,

$$\left(\frac{\partial\mu_i}{\partial n_i}\right)_T = \frac{k_B T}{n_i} \tag{8}$$

where k_B is the Boltzmann constant. According to the Maxwell relation,

$$\left(\frac{\partial\mu_i}{\partial T}\right)_{n_i} = -\left(\frac{\partial S}{\partial n_i}\right)_T = -s_i \tag{9}$$

where S is the entropy of the system, and s_i is the partial molar entropy. Finally, Eq. (8) and Eq. (9) are substituted into Eq. (7):

$$\nabla\tilde{\mu}_i = \frac{k_B T}{n_i}\nabla n_i - s_i\nabla\mathrm{T} + q_i\nabla V \tag{10}$$

Next, L_{ii} and L_{iQ} are expressed as[10]:

$$L_{ii} = \frac{n_i D_i}{k_B} \tag{11}$$

$$L_{iQ} = L_{Qi} = T\bar{\bar{S}}_i L_{ii} \tag{12}$$

where $\bar{\bar{S}}_i$ is named the transported entropy[11]. And Eastman entropy of transfer[12] is defined as:

$$\hat{S}_i = \bar{\bar{S}}_i - s_i \tag{13}$$

Substituting Eq. (10-13) into Eq. (6), we get the expression of the ionic flow related to the electrochemical potential and the temperature gradient as follows:

$$J_i = -D_i\left(\nabla n_i + \frac{q_i n_i}{k_B T}\nabla V + \frac{\hat{S}_i n_i}{k_B T}\nabla T\right) \tag{14}$$

For our materials, we tested the ionic thermoelectric signal in an open circuit. In open circuit condition, there is no net current of ions. Therefore, we have:

$$\sum_i q_i J_i = -\sum_i D_i \left(q_i \nabla n_i + \frac{q_i^2 n_i}{k_\mathrm{B} T} \nabla V + \frac{q_i \hat{S}_i n_i}{k_\mathrm{B} T} \nabla T \right) = 0 \tag{15}$$

Near equilibrium, $n_i(r) = n_i^0 + \delta n_i(r) \approx n_i^0$. Eq. (15) can be simplified as:

$$\sum_i \left(\frac{q_i^2 n_i^0 D_i}{k_\mathrm{B} T} \nabla V + \frac{q_i n_i^0 \hat{S}_i D_i}{k_\mathrm{B} T} \nabla T \right) = 0 \tag{16}$$

Then, the Seebeck coefficient derived as:

$$S_\mathrm{t} = -\frac{\nabla V}{\nabla T} = \frac{\sum_i \, q_i n_i^0 \hat{S}_i D_i}{\sum_i \, q_i^2 n_i^0 D_i} \tag{17}$$

Because ionic compounds have a net charge of zero, i.e.:

$$\sum_i q_i n_i^0 = 0 \qquad q_+ n_+^0 = -q_- n_-^0 \tag{18}$$

Therefore, we have:

$$S_\mathrm{t} = \frac{D_+ \hat{S}_+ - D_- \hat{S}_-}{e(D_+ + D_-)} \tag{19}$$

When there is no electric field and no concentration gradient, the equation can be simplified as:

$$J_i = -n_i \frac{D_i \hat{S}_i}{k_\mathrm{B} T} \nabla T = -n_i v_i^T \tag{20}$$

where $v_i^T = \frac{D_i \hat{S}_i}{k_\mathrm{B} T} \nabla T$ is defined as thermodiffusion velocity. Combined with the Einstein's relation for thermodiffusion, the thermal mobility μ_i^T is defined as：

$$\mu_i^T = \frac{D_i \hat{S}_i}{k_\mathrm{B} T} \tag{21}$$

Therefore, we can see that the Seebeck coefficient is determined by the difference in thermal mobilities of the cations and anions. The thermal mobility is determined by diffusion coefficient

D_i and the Eastman entropy of transfer $\hat{S}_i$.

$\hat{S}$ is essentially related to the interaction between ions and the surrounding polymers. The relevant discussions are as follows.

5. Interactions between ionic liquids and FE polymers

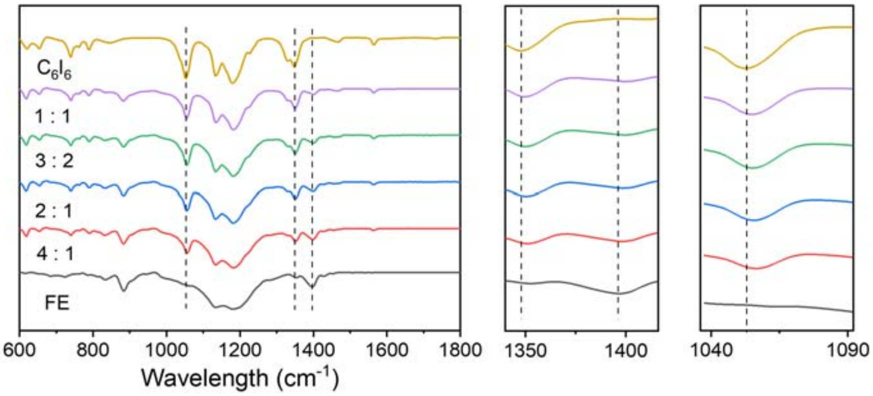

Supplementary Fig. 23 | FTIR spectroscopy of FE-C6I6s with different contents.

For $TFSI^-$ anion[13,14], the typical S=O antisymmetric stretching band at 1348 cm^{-1} shifted to 1352 cm^{-1}. And the N-S antisymmetric stretching band peaked at 1052 cm^{-1} shifted to 1057 cm^{-1} (Supplementary Fig. 23). The blue shift of the two bands indicated that S=O and N-S bonds were stronger. Relative to pure C6I6, there is a degree of separation of cations from anions in the composites. Because the cation can be interacted with fluorinated polymers with a partial negative charge, leading to more negative charges on the N atom of TFSI anions. The interaction is independent of the content. Small shifts prove weak interactions. The interactions are defined as ion-dipole interactions[15-17]. The interaction is non-directional and dynamic, adapting to the various movements of the polymer without being disrupted. This is one of the reasons why the movement

of ions in the material has been able to remain stable.

In addition, it was observed that the CH_2 wagging band shifted from 1396 to 1402 cm^{-1} (Supplementary Fig. 23), indicating a weaker interaction between the H atoms and the F atoms on the polymer chains[18]. In other words, the distance between the polymers increased because of the interactions with the cations. The phenomenon is the result of the plasticizing effect.

Apart from the changes mentioned above, the rest peaks of the composites are unchanged compared to the pure component. Therefore, there are no strong interactions between ionic liquids and polymers, which cannot be repaired after damage. The plasticizing effect can also be observed in the mechanical properties.

6. Mechanical properties affected by ionic liquids

As the ionic liquid content increases, the glass transition temperature (T_g) gradually decreases from -27 °Cto 40 °C, and the Young's modulus decreases from 0.92 MPa to 0.11 MPa (Supplementary Table 2). This is typical of the plasticizing effect, where the ionic liquid is the plasticizer. In addition to this, the different types of ionic liquids have different effects on the composites. From the Young's modulus of the complexes at the same ratio, EMITFSI exhibits similar properties to HMITFSI, and C6I6 is consistent with C1I6 (Supplementary Table 2). This is the effect of the entropy effect on the Young's modulus of the material. The lower the entropy of the system, the higher the Young's modulus, i.e. the greater the resistance to external stresses[19].

Among the composites, FE-C6I6 (2:1) has the best toughness and high Young's modulus due

to the absence of defects and the moderate content of ionic liquids (Supplementary Fig.5, 6).

Supplementary Table 2. Mechanical properties of the composites

	Young's Modulus (MPa)	Toughness (MJ/m^3)	T_g (°C) *
FE-C6I6 (4:1) **	0.92±0.02	0.78±0.05	-27
FE-C6I6 (2:1)	0.71±0.02	0.98±0.05	-34
FE-C6I6 (3:2)	0.27±0.02	0.50±0.04	-38
FE-C6I6 (1:1)	0.11±0.01	0.10±0.02	-44
FE-EMITFSI (2:1)	0.27±0.02	0.51±0.03	-39
FE-HMITFSI (2:1)	0.33±0.02	0.42±0.03	-38
FE-C1I6 (2:1)	0.70±0.01	0.65±0.03	-28

*The glass transition temperature is measured by DSC. **The content in parentheses is the mass ratio (FE : XX).

7. Ionic thermoelectric properties affected by ionic liquids

Supplementary Table 3. Electronic properties of the composites.

	Ionic conductivity (S/cm)	Seebeck coefficient (mV/K)
FE-C6I6 (4:1) *	4.45×10^{-7}	0.25
FE-C6I6 (2:1)	4.86×10^{-6}	0.51
FE-C6I6 (3:2)	3.28×10^{-5}	0.60
FE-C6I6 (1:1)	2.89×10^{-4}	0.75
FE-EMITFSI (2:1)	3.65×10^{-5}	0.4
FE-HMITFSI (2:1)	1.48×10^{-5}	0.41
FE-C1I6 (2:1)	2.77×10^{-6}	0.18

*The content in parentheses is the mass ratio (FE : XX).

The ionic conductivity and ionic Seebeck coefficient are both related to ion concentrations (**Supplementary Table 3**). Higher ionic conductivity and ionic Seebeck coefficient can both lead to higher ionic thermoelectric figure of merit. In this manuscript, the temperature sensing ability

of our device is mainly related to the ionic Seebeck coefficient. And there is no direct positive or negative correlation between ionic conductivity and ionic Seebeck coefficient. Thus, ionic conductivity is not the first priority in this work.

To optimize the performance of our device, we investigated how the influence of ion liquid types and amount on Seebeck coefficient (**Supplementary Fig. 24**) and mechanical property. Finally, we choose C6I6 as the mobile ions and FE/IL ratio of 2:1 to achieve the balance between temperature detection sensitivity and stability underwater.

The Seebeck coefficient is not significantly related to the amount of ionic liquids (Supplementary Fig. 24). This may be related to the weak interactions between ionic liquids and polymers.

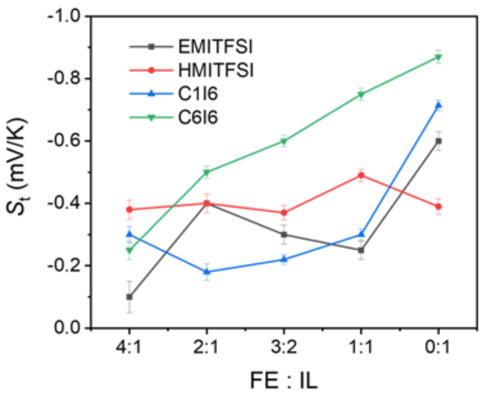

Supplementary Fig. 24 | Seebeck coefficient of the composites and pure ionic liquids without water.

Although pure ionic liquids can be layered when mixed with water, the complex flow makes it impossible to distinguish between different temperature gradients due to the irregularity of its signal. The opposite direction of the temperature gradient does not determine the positive or

negative signal. For FE-EMITFSI/HMITFSI/C1I6 (2: 1), the signal is equally complex with poor repeatability after a week of immersion in water. Combined with Fig.2 and Supplementary Table 2, we chose FE-C6I6 (2: 1) for our study.

8. Parameters for finite element analysis (FEA)

Parameters of water include: coefficient of thermal expansion, bulk viscosity, kinematic viscosity, ratio of specific heat, constant pressure heat capacity, density, and thermal conductivity. And they are dependent on temperature. The values or functions are collected in the library of COMSOL. Other parameters are collected in Supplementary Table 2.

Supplementary Table 2. Mechanical properties of the composites

Parameter	Value	Description
L_{channel}	100 m	Channel length
W_{channel}	10 m	Channel width
H_{channel}	10 m	Channel height
L_{fish}	2 m	Fish length
W_{fish}	1 m	Fish width
H_{fish}	1 m	Fish height
L_{heater}	2 cm	Heater length
W_{heater}	3 cm	Heater width
H_{heater}	0.17 mm	Heater thickness
$T_{0(\text{fish})}$	283.15K	Water temperature (fish)
$T_{0(\text{heater})}$	293.15K	Water temperature (heater)

9. Calibration of measured temperatures

The values of the various thermometers are determined by the temperature scale. A temperature scale is a standard measure of temperature established to ensure uniformity and

accuracy of temperature measurements. Therefore, we need a standard thermometer to determine the temperature values.

In this paper, the standard thermometers used to determine the temperature are platinum resistance thermometers (PT100) as well as IR camera (FLIR X6530sc). But the resolution of both does not reach 0.001K. So we use two methods to confirm the currently measured temperatures:

1) We use standard thermometers to measure the temperature change on our device. In our experiment we use Peltier elements to control device temperature and build temperature difference. For horizontal-structured device, two Peltier elements were used to heat and refrigerate, separately. For vertical-structured device, one Peltier element was used to heat or refrigerate the device bottom. For example, the upside and downside temperature of vertical-structured device (**Figure R2**) were both measured with PT100 while the Peltier element working current was programmable changed. Further we can get a good linear relationship between temperature difference on device and working current of the Peltier elements. And lesser temperature difference can be constructed by precisely changing Peltier element current.

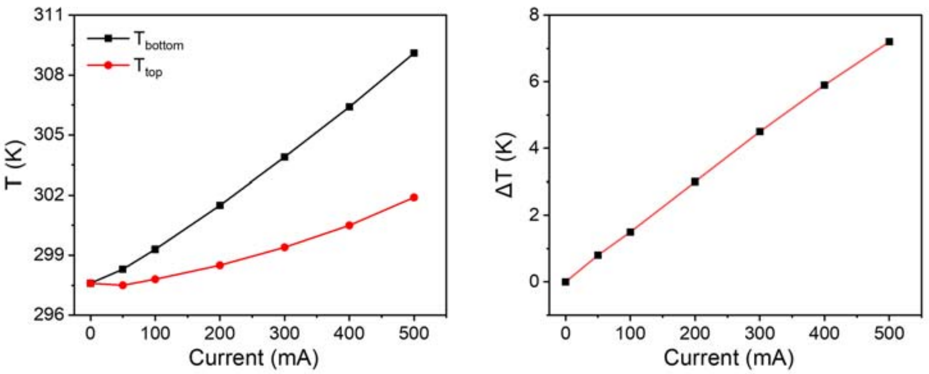

Supplementary Fig. 25 | Calibration of temperatures at both ends of a vertical structure and the

operating curve between the operating current of Peltier elements and the temperature difference.

2) By further measure the voltage change on hot/cold sides, we can calculate the Seebeck coefficient of FE-C6I6 by establishing the relationship between the voltage variation and the temperature difference (above 0.1 K) (**Figure R3**). The Seebeck coefficient maintain good consistency not only for high (above 0.1 K) and low (below 0.1 K) temperature differences, but also for horizontal and vertical structured devices. The temperature corresponding to the currently measured voltage is confirmed.

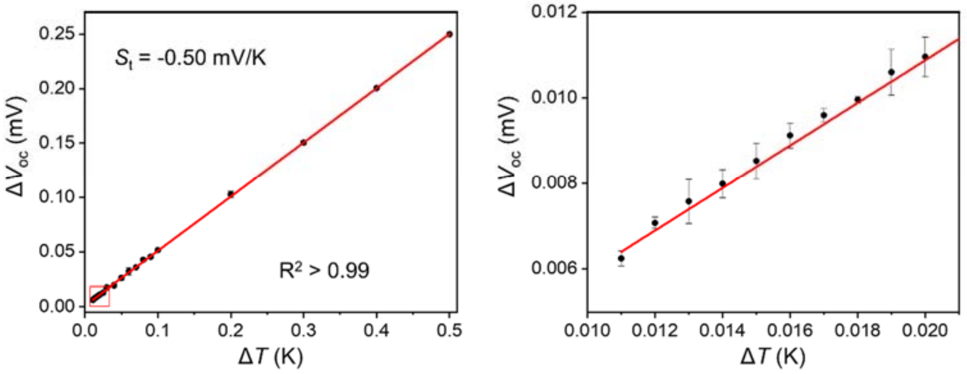

Supplementary Fig. 26 | Dependence of ΔV_{oc} on ΔT . The material is exposed to air. The resolution is 0.001 K. Error bars show s.d., n = 3.

10. Temperature resolution in saturated solution of NaCl

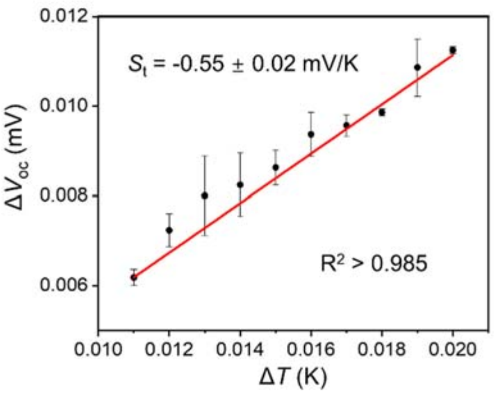

Supplementary Fig. 27 | Dependence of ΔV_{oc} on ΔT . A saturated solution of NaCl was used in the test. The resolution is 0.001 K. Error bars show s.d., n = 3.

11. Thermal conductivity of FE-C6I6

Supplementary Table 3. Thermal conductivity (κ) of FE-C6I6 at different temperature.

Temperature (°C)	-20.77	-0.77	19.23	39.23	59.23
κ (W m^{-1} K^{-1})	0.114	0.124	0.135	0.147	0.159

Supplementary Reference

1 Kitov, P. I. & Bundle, D. R. On the nature of the multivalency effect: a thermodynamic model. *J. Am. Chem. Soc.* **125**, 16271-16284, doi:10.1021/ja038223n (2003).

2 Berthier, L., Ozawa, M. & Scalliet, C. Configurational entropy of glass-forming liquids. *J. Chem. Phys.* **150**, 160902, doi:10.1063/1.5091961 (2019).

3 Seki, K. & Bagchi, B. Relationship between entropy and diffusion: A statistical mechanical derivation of Rosenfeld expression for a rugged energy landscape. *J. Chem. Phys.* **143**, 194110, doi:10.1063/1.4935969 (2015).

4 Riess, J. G. Understanding the fundamentals of perfluorocarbons and perfluorocarbon emulsions relevant to in vivo oxygen delivery. *Artif. Cells Blood Substit. Immobil. Biotechnol.* **33**, 47-63, doi:10.1081/bio-200046659 (2005).

5 Heinz, H. *et al.* Nanoparticle decoration with surfactants: Molecular interactions, assembly,

and applications. *Surf. Sci. Rep.* **72**, 1-58, doi:10.1016/j.surfrep.2017.02.001 (2017).

6 You, I. *et al.* Artificial multimodal receptors based on ion relaxation dynamics. *Science* **370**, 961-965, doi:10.1126/science.aba5132 (2020).

7 Grahame, D. C. The electrical double layer and the theory of electrocapillarity. *Chem. Rev.* **41**, 441-501, doi:10.1021/cr60130a002 (1947).

8 Han, C. G. *et al.* Giant thermopower of ionic gelatin near room temperature. *Science* **368**, 1091-1098, doi:10.1126/science.aaz5045 (2020).

9 Chen, B. *et al.* Giant negative thermopower of ionic hydrogel by synergistic coordination and hydration interactions. *Sci. Adv.* **7**, eabi7233, doi:10.1126/sciadv.abi7233 (2021).

10 Bonetti, M., Nakamae, S., Roger, M. & Guenoun, P. Huge Seebeck coefficients in nonaqueous electrolytes. *J. Chem. Phys.* **134**, 114513, doi:10.1063/1.3561735 (2011).

11 J. N. Agar, in Advances in Electrochemistry and Electrochemical Engineering, edited by P. Delahay (Interscience, New-York, 1963), Vol. 5, Chap. 2.

12 Eastman, E. D. Theory of the Soret Effect. *J. Am. Chem. Soc.* **50**, 283-291, doi:10.1021/ja01389a007 (1928).

13 Talaty, E. R., Raja, S., Storhaug, V. J., Dölle, A. & Carper, W. R. Raman and Infrared Spectra and ab Initio Calculations of C2-4MIM Imidazolium Hexafluorophosphate Ionic Liquids. *J. Phys. Chem. B* **108**, 13177-13184, doi:10.1021/jp040199s (2004).

14 Rey, I. *et al.* Spectroscopic and Theoretical Study of (CF3SO2)2N-(TFSI-) and (CF3SO2)2NH (HTFSI). *J. Phys. Chem. A* **102**, 3249-3258, doi:10.1021/jp980375v (1998).

15 Cao, Y. *et al.* A Transparent, Self-Healing, Highly Stretchable Ionic Conductor. *Adv. Mater.* **29**, 1605099, doi:10.1002/adma.201605099 (2017).

16 Cao, Y. *et al.* Self-healing electronic skins for aquatic environments. *Nat. Electron.* **2**, 75-82, doi:10.1038/s41928-019-0206-5 (2019).

17 Zhang, Y. *et al.* Highly Transparent, Underwater Self-Healing, and Ionic Conductive Elastomer Based on Multivalent Ion–Dipole Interactions. *Chem. Mater.* **32**, 6310-6317, doi:10.1021/acs.chemmater.0c00096 (2020).

18 Li, Z., Su, G., Gao, D., Wang, X. & Li, X. Effect of Al2O3 nanoparticles on the electrochemical characteristics of P(VDF-HFP)-based polymer electrolyte. *Electrochim. Acta* **49**, 4633-4639, doi:10.1016/j.electacta.2004.05.018 (2004).

19 Weis-Fogh, T. Molecular interpretation of the elasticity of resilin, a rubber-like protein. *J. Mol. Biol.* **3**, 648-667, doi:10.1016/s0022-2836(61)80028-4 (1961).
